# Structures of a Mobile Intron Retroelement Poised to Attack Its Structured DNA Target

**DOI:** 10.1126/science.abq2844

**Published:** 2022-11-10

**Authors:** Kevin Chung, Ling Xu, Pengxin Chai, Junhui Peng, Swapnil C. Devarkar, Anna Marie Pyle

**Affiliations:** 1 Department of Molecular Biophysics and Biochemistry, Yale University, New Haven, CT 06511, USA.; 2 Department of Molecular, Cellular and Developmental Biology, Yale University, New Haven, CT 06511 USA.; 3 Howard Hughes Medical Institute, Chevy Chase, MD 20815, USA.; 4 Laboratory of Evolutionary Genetics and Genomics, The Rockefeller University, New York, NY 10065, USA.

## Abstract

Group II introns are ribozymes that catalyze their self-excision and function as retroelements that invade DNA. As retrotransposons, group II introns form ribonucleoprotein complexes that roam the genome, integrating by reversal of forward splicing. Here we show that retrotransposition is achieved by a tertiary complex between a structurally elaborate ribozyme, its protein mobility factor, and a structured DNA substrate. We solved cryoEM structures of an intact group IIC intron-maturase retroelement poised for integration into a DNA stem-loop motif. By visualizing the RNP before and after DNA targeting, we show that it is primed for attack, and fits perfectly with its DNA target. This study reveals design principles of a prototypical retroelement and reinforces the hypothesis that group II introns are ancient elements of genetic diversification.

## One-Sentence Summary

Group II intron RNPs are elaborately organized retroelements that can recognize the shape and sequence of their structured DNA target.

**Figure 1.**
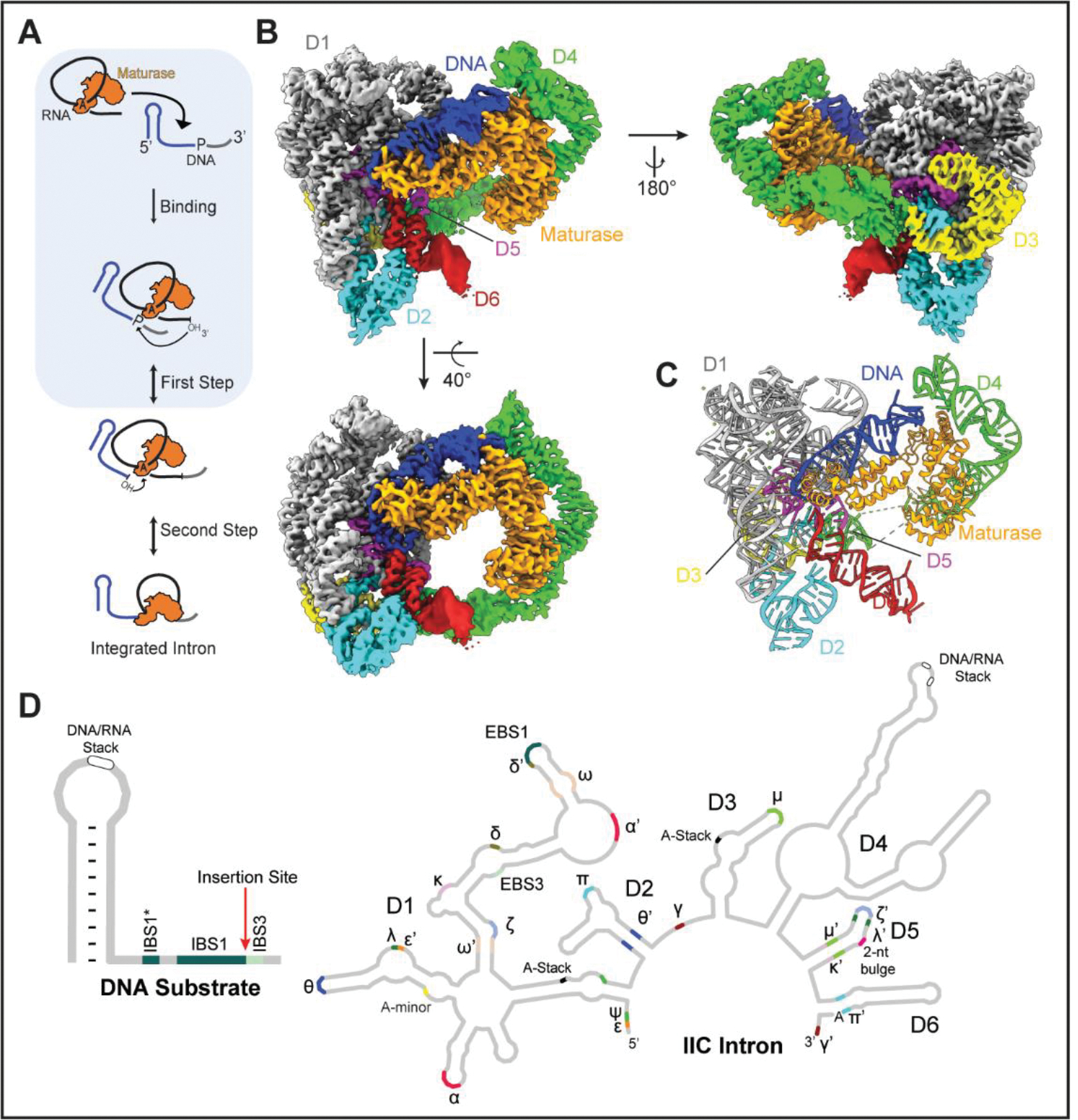
CryoEM Reconstruction of a Group II Intron Retroelement **(A).** Cartoon of the reverse splicing reaction. **(B).** Composite cryoEM map of the holoRNP with bound DNA. **(C).** Molecular model of the group II intron retroelement. **(D).** Secondary structure cartoon and tertiary interactions of the holoRNP.

Group II introns are self-splicing retroelements that have played a key role in shaping eukaryotic genomes as the ancestors of spliceosomal introns and non-LTR retroelements (1). They remain important for gene expression in plants, fungi, yeasts, and many bacteria (2, 3). Group II introns encode a specialized reverse transcriptase (maturase or intron-encoded protein (IEP)), that binds its parent intron and facilitates self-splicing, releasing a well-folded lariat ribonucleoprotein (RNP) complex (4). The liberated RNP functions as a retrotransposon, targeting DNA that contains spliced exon junction sequences and inserting via a two-step transesterification reaction known as reverse splicing (Fig. 1A) (5). The resulting DNA-RNA chimera is copied into cDNA by the reverse transcriptase (RT) activity of the multifunctional IEP in a process known as target primed reverse transcription (TPRT) (6). Host repair pathways complete the downstream DNA copy-and-paste steps needed to achieve total intron integration (4).

There are three main classes of group II introns, IIA, IIB and IIC, which share a conserved secondary structure and a similar tertiary organization around a ribozyme active site (7). Group IIC introns are an ancient class of bacterial introns that recognize both the sequence and three-dimensional structure of their DNA recognition sites (8). Unlike their larger IIA and IIB counterparts, group IIC introns are almost completely dependent on their IEPs to facilitate intron excision through lariat formation, thereby forming the functional RNP that serves as the minimal element for retrotransposition (8). Compared to their more evolved counterparts, group IIC IEPs lack an endonuclease domain for generating TPRT primers and instead exploit the lagging strands at DNA replication forks (4).

Recent structural and biochemical studies of IIA and IIB introns have provided important insights into strategies for IEP recognition of intron RNA (9–11). However, available RNP structures have not revealed a specific mechanistic role for the IEP during RNP assembly, DNA recognition, or chemical catalysis. At present, the mechanism by which group IIC introns recognize DNA structures, and not just DNA sequences, remains unclear. Furthermore, there are no available structures of the free RNP retroelement before it has bound DNA. These open questions preclude a clear understanding of group II intron retrotransposition and its evolutionary role in shaping modern genomes. To address these problems, we solved cryoEM structures of a group IIC intron retroelement poised to undergo the first step of reverse splicing. Our findings have important implications for understanding mechanistic function of the spliceosome and non-LTR retrotransposons, which continue to remodel and impact the function of human genomes (12, 13).

## Results

### Overall Architecture of an Ancient Group II Intron Retroelement

To investigate the mechanism of DNA insertion, we captured a group II intron retroelement prior to the first step of reverse-splicing into DNA (Fig. 1A). We first conducted *in vitro* splicing reactions of the IIC *Eubacterium rectale (E.r.)* (14) intron in complex with its encoded maturase (MarathonRT) (15, 16) and purified the reaction mixture to obtain a branched lariat-maturase complex (fig. S1, A to C). Purity and stability of this RNP complex were assessed using biophysical methods: sedimentation velocity analytical ultracentrifugation and size exclusion chromatography coupled to multi-angle light scattering indicated that the sedimentation coefficient and molecular mass of the RNP were larger compared to the those of the individual lariat or maturase components, suggesting complex formation (fig S1, D to F). To visualize the retroelement in action, we introduced a desthiobiotin-tagged DNA substrate to the intron-maturase RNP and isolated ternary complexes by affinity purification on an avidin column (fig S2). The purified elution fraction was vitrified on grids and the holoenzyme molecules appeared as monodisperse particles on cryoEM micrographs, thereby allowing structure determination (fig. S2, B to D and fig. S3).

Initial data analysis suggested preferred orientation of the sample, so a tilted data collection strategy was required to obtain additional projection views (fig. S3). After further classification and focused refinement, we obtained a 2.8Å resolution cryoEM structure of the *E.r*. group IIC intron in complex with its specific IEP and DNA target (Fig. 1, B and C, and fig. S4 and S5), thereby revealing the state immediately prior to the first step of reverse splicing (Fig. 1A). The overall high-resolution 3D reconstruction was of sufficient quality to permit modelling of individual nucleotides (movie S1) and metal ions. The catalytic core formed by D5, the lariat branchpoint, EBS-IBS (exon binding site-intron binding site) sequences and the protein thumb and DNA binding domain (DBD) were resolved to < 3Å.

The overall structure reveals a compact assembly of intron RNA and maturase protein that is closely associated with the DNA substrate through a network of interactions (movie S2). The intron core adopts a similar fold to that of intron structures derived from truncated and modified constructs (17, 18). Tertiary interactions identified in previous group II introns are present, along with several additional novel interactions that are observed in this full-length intron construct that contains all six intron domains (Fig. 1D). The fold of the maturase resembles that of previously studied IIC proteins (15, 19), although the thumb and DNA binding domains are now clearly resolved (Fig. 1C and fig. S5). The bound DNA contains a short spacer, the intron insertion site and a 5’ stem-loop motif unique to group IIC introns (20, 21) (Fig. 1D).

### Features of the Catalytic RNP Core

**Figure 2.**
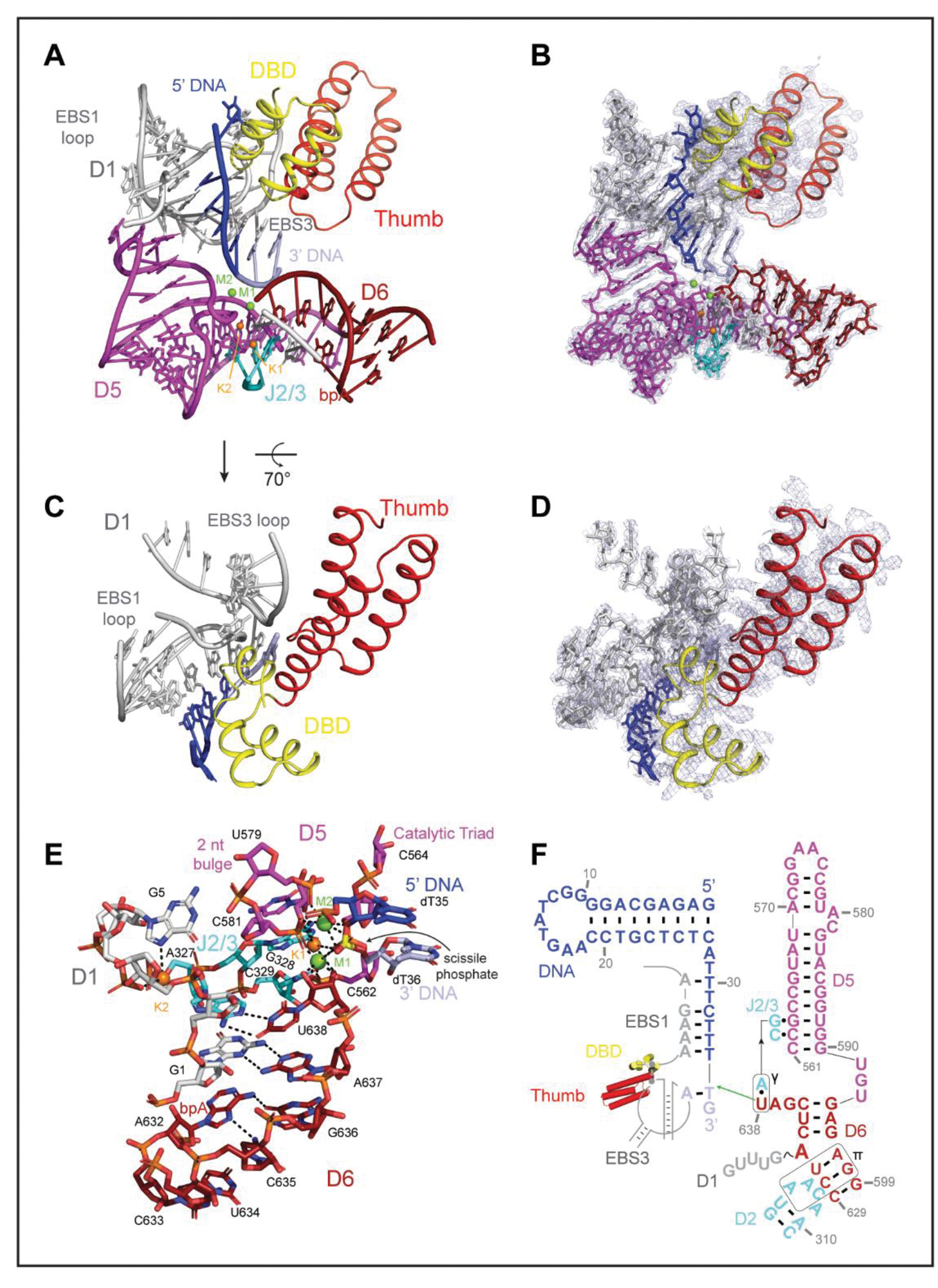
Architecture of an Intron Retroelement Active Site **(A-B).** Organization of the holoRNP core domains. **(C-D)** The maturase DBD and thumb domains stabilize the DNA recognition loops. **(E-F).** Model and secondary structure schematic of the intron retroelement prior to the first step of retrotransposition.

Despite extensive efforts, a complete group II intron holoenzyme active site had not yet been visualized. In this work, we capture the complete ribozyme core architecture, which includes hallmark elements identified in earlier biochemical and structural studies (8, 22). For example, we see that the 2’−5’ lariat linkage, between the first intron nucleotide (G1) and the branchpoint A (A632), is a crucial structural motif for organizing the ribozyme core. The branch-site actively engages the 3’ end of the intron (G1-A637 pairing), helping to position the terminal nucleotide (U638) for nucleophilic attack on DNA (Fig. 2, A and B) (23, 24). Facilitating this process, U638 base-pairs with A327 to form the γ-γ’ interaction (Fig. 2, E and F) (23, 24). The adjacent G328 and C329 nucleotides of the J2/3 linker form major-groove base triples with C562 and G563 (fig. S6A), giving rise to the catalytic triplex common to all group II introns and the spliceosome (25, 26). The 2-nt bulge (A580, C581) and catalytic triad (C562, G563, C564) in D5, along with U638, all serve to coordinate catalytic magnesium ion M1, placing it between the nucleophilic 3’ OH and scissile phosphate, in an arrangement poised for the first step of reverse splicing (Fig 2, A, B and E) (17). A second magnesium ion, M2, is located 3.9Å away from M1, consistent with the two-metal ion catalysis mechanism (Fig. 2, A, B and E) (17, 27). We identified two additional, unambiguous densities at positions previously assigned to monovalent ions K1 and K2 in studies that employed anomalous scattering to establish sites of stable K^+^ binding (26, 28). In that case, as in this instance, NH_4_^+^ can functionally substitute for K^+^ at these same positions (Fig. 2, A, B and E). The specific coordination and placement of these monovalent ions is essential for positioning the catalytic divalent metal ions, forming a reactive, heteronuclear metal ion cluster. Several tertiary interactions stabilize the periphery of the catalytic core, with D3 and a novel A-stacking interaction bracing the backside of the D5 helix (μ-μ’) (fig. S6, B to C). D2 contacts D6 (π-π’) to hold the lariat in place (23, 29) (fig. S6D). Although many of these active site elements have been observed independently, in linear introns or in introns of other classes, they have not been simultaneously captured in a single structure until now, thereby demonstrating that these active site elements function in concert and are conserved. The *E.r.* holoenzyme structure provides a detailed view of a complete, reactive intron catalytic core (movie S3).

Close inspection of the active site reveals structural interdependence between the intron and its encoded maturase. Within the active site, the intron RNA forms short base pairings with its target DNA via the EBS-IBS interactions (EBS1-IBS1 and EBS3-IBS3) (Fig. 2, A, B, E and F). These otherwise unstable short pairing interactions are buttressed and positioned by the maturase, which presses the middle α-helices of the DBD and the third α-helix of the thumb domain against the EBS1 and EBS3 recognition loops respectively (Fig. 1B and 2C), rigidifying them and helping to form a central cavity for engagement with DNA (Fig. 2, C and D). These findings establish that the retroelement core does not consist solely of RNA, rather it is a collaborative, ribonucleoprotein active site. The new roles we observe for the maturase thumb and DBD help to explain the strong maturase dependence for both RNA splicing and intron integration, particularly *in vivo*, and they highlight the symbiotic relationship between the intron RNA and its protein cofactor, which are known to have co-evolved (30, 31).

### Functional Coordination Between RNA and Protein

**Figure 3.**
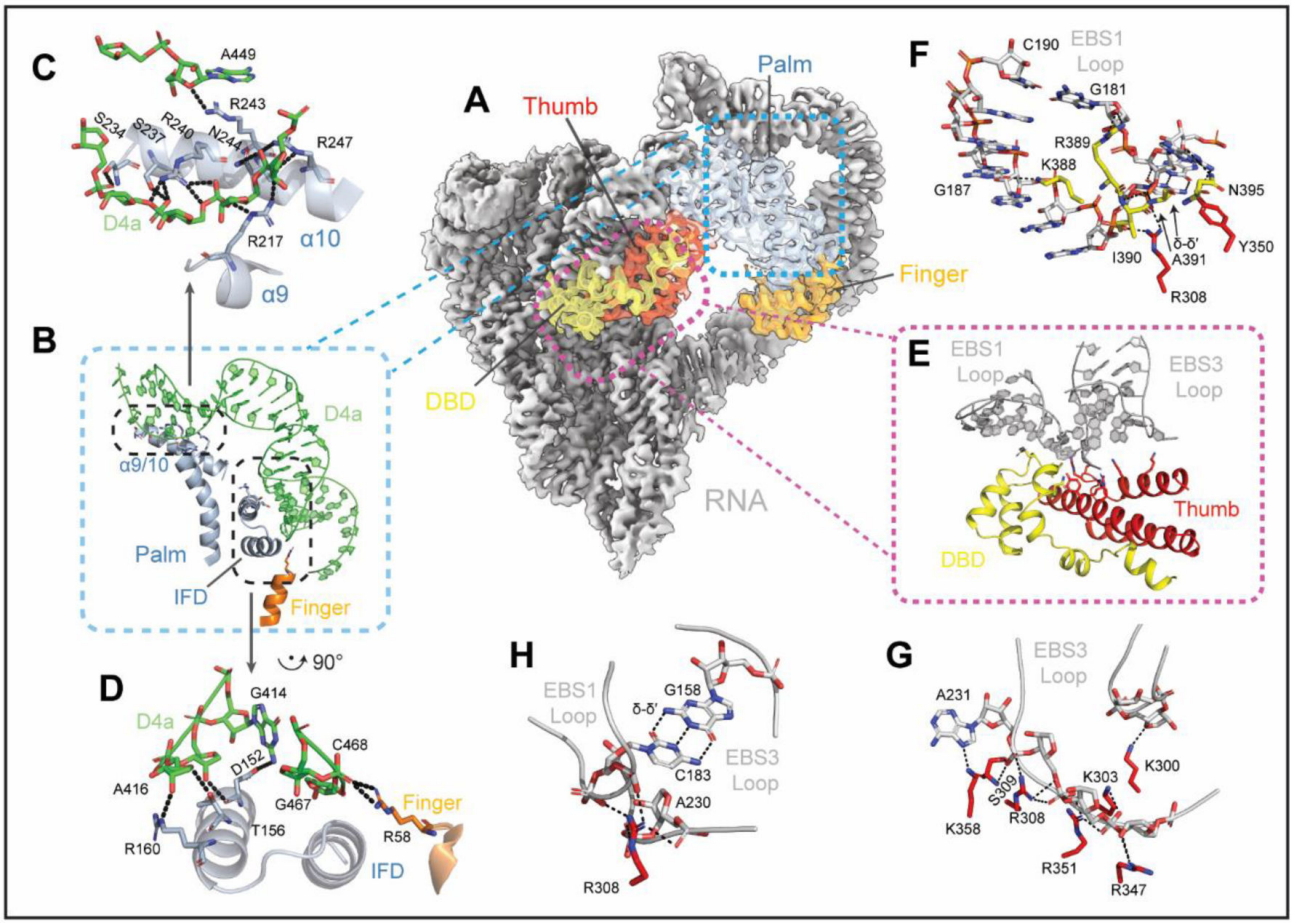
Mechanism of Maturase Facilitated Ribozyme Catalysis **(A).** Protein positioning within the retroelement composite map. **(B).** Protein-D4a contact points. **(C-D).** Interactions that form the RNA-protein anchor points. **(E).** Protein stabilization of EBS1 and EBS3 loops. **(F-G).** Amino acids that rigidify the EBS1 and EBS3 loops **(H).** R308 joins EBS1 and EBS3 together.

A striking feature of the retroelement holoenzyme is the expansive D4 arm, which extends far from the core and then curves around to cradle the maturase (Fig. 3A). D4a, the high affinity maturase-binding subdomain (15, 32), forms two anchor points with basic protein surfaces (Fig. 3, A and B) (33). At the first anchor point, residues extending from the protein (R58, D152, T156, and R160) interact with RNA phosphate and ribose oxygens, securing the insertion helix within the finger domain (IFD) of the IEP against the minor groove interface in the middle of the long D4a hairpin (Fig. 3, B and C). A sharp turn places the distal portion of the D4a subdomain between α-helices 9 and 10 of the protein, where largely basic residues (R217, S234, S237, R240, R243, N244 and R247) approach the RNA backbone from either side, fastening the palm to the D4a arm (Fig. 3, B and D). In contrast with other group II RNPs, the surface of the finger domain (RT0) is not utilized for RNA recognition (9, 10, 15) (fig. S7).

The distinct intron-maturase recognition strategy places the maturase thumb and DBD next to the intron core, allowing the protein to participate in catalysis by rigidifying the active-site (Fig. 3, A and E). The thumb and DBD grasp the EBS1 and EBS3 loops, directly coordinating substrate recognition elements within the retroelement active site (Fig. 3E). One approach of this strategy involves locking EBS nucleotides into a conformation conducive for substrate binding (i.e. K388 with G187O6 of EBS1 and K358 with A231N7 of EBS3) (Fig. 3, E to G). A secondary tactic includes immobilizing the EBS3 phosphate backbone through interactions with a multitude of basic residues on the protein thumb (K300, K303, S309, R347 and K358) (Fig. 3G). A third strategy consists of DBD amino acids (Y350, R389, N395 and main chain amines of I390 and A391), stabilizing the turn in EBS1 and enabling the formation of δ-δ’, thereby reinforcing this single base pair interaction (C183 with G158) that bridges the EBS loops. (Fig. 3F). Interestingly, R308 of the protein thumb provides additional stabilization by simultaneously coordinating the phosphate backbone of EBS1 and 3 (through C183 and A230) (Fig. 3H). These interactions demonstrate a specific mechanistic role for the maturase protein during catalysis, showing that it promotes proper formation of multiple active site components (34). These findings reveal the inextricable, functional coordination of intron and protein during the mechanism of splicing and retrotransposition.

### Tertiary Interactions with a Structured DNA

**Figure 4.**
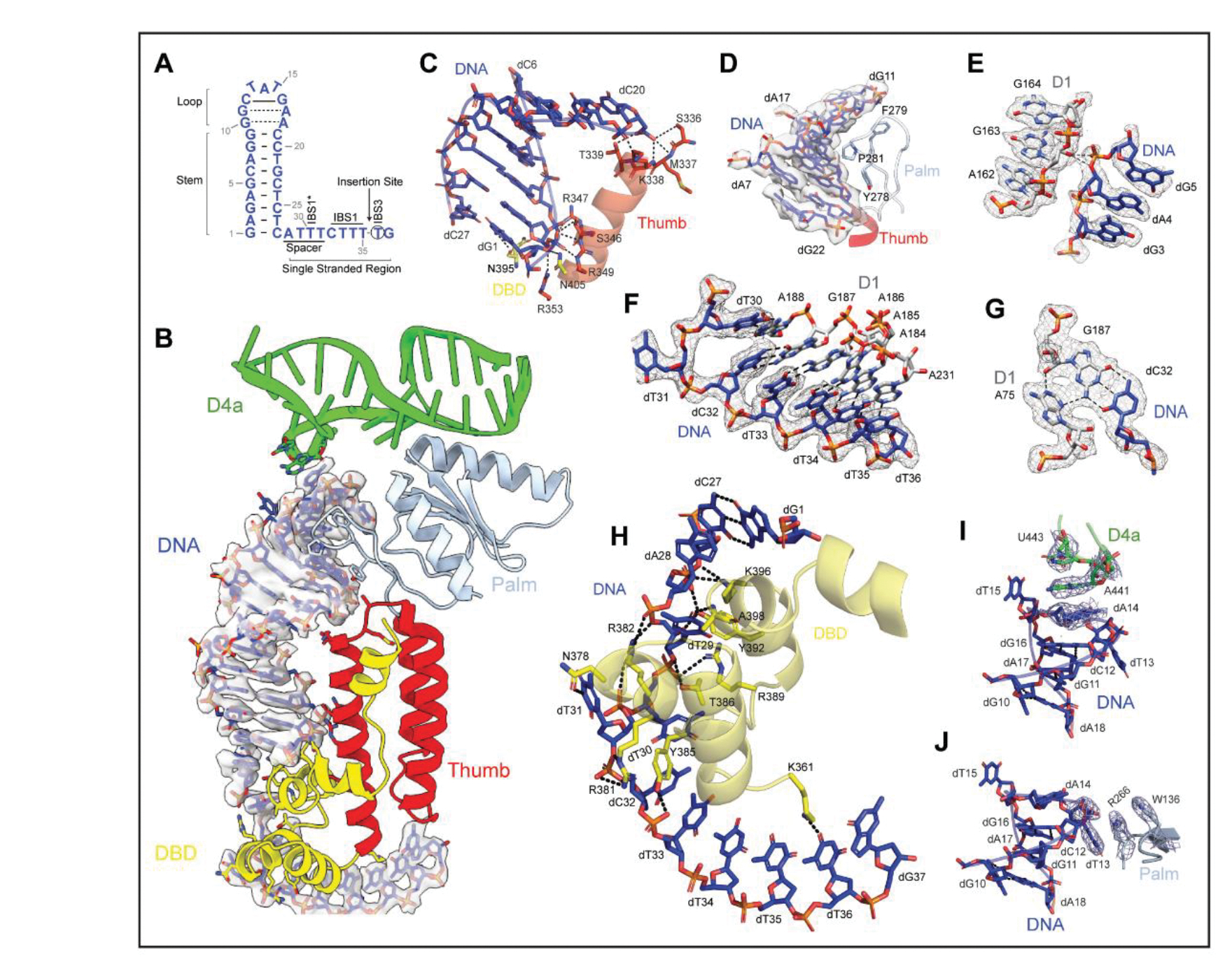
Shape and Sequence Recognition of a DNA Target **(A).** Secondary structure of the DNA target. **(B).** Interactions of the structured DNA with holoRNP. **(C).** Protein contacts with DNA helical stem. **(D).** Fit of the DNA groove against the protein palm linker. **(E).** DNA and D1 backbone interactions. **(F).** EBS-IBS base pairing interactions. **(G).** A stabilizing A-minor tertiary interaction. **(H).** Interactions between protein and single stranded DNA. **(I-J).** Intermolecular stacking interactions between DNA and **(I)** RNA nucleotides and **(J)** protein residues.

Some of the most remarkable features of this structure involve the DNA target and the unusual strategies for molecular recognition by the holoenzyme. Here we show that the DNA is recognized through a combination of shape-selectivity and base-specific interactions (movie S4), only a few of which involve canonical WC pairing. The DNA itself has distinct structural features that support this recognition strategy. Most prominent is an unusual, structurally conserved, DNA stem, which is composed of a short helix [9 base pairs (bp)] that is capped by an undertwisted duplex comprised of two noncanonical G-A DNA base-pairs and a G-C base pair. Together, these extend the DNA stem to 12 bp, which approximates the consensus stem length for IIC insertion targets. The terminal DNA loop serves as a stacking platform for long-range interactions. Adjacent to the DNA stem is a short spacer, which is followed by IBS1 nucleotides and the IBS3 nucleotide that flanks the DNA insertion site (Fig. 4A and B).

The DNA stem lies in a cleft that is formed by regions of both the protein (DBD and thumb) and the intron RNA (D1d and D4a). Two clusters of amino acids along the third α-helix of the protein thumb domain anchor the DNA stem by making contacts at both ends of the DNA helix, at positions separated by approximately one helical turn. The first cluster (S346, R347, R349, R353, N395, and N405) secures the base of the stem through contacts with dG1 and dA2 (Fig. 4C). The second group (S336, M337, K338, and T339) appears to locally deform the top base pairs of the stem at dC20 and dT21 (Fig. 4C). This is the result of a striking DNA-protein interaction network that involves insertion of a prolyl-aromatic loop into the distorted, widened minor groove at the tip of the DNA stem. The complementary fit of this peptide loop is mediated by interactions between largely buried side chains (Y278, F279 and P281) and the methylene edges of DNA sugar moieties (Fig. 4D). These protein-DNA interactions are supported by contacts between the DNA and RNA backbone residues (dA4O3’ and dG5OP1 with G163 2’OH), reminiscent of ribose-zipper interactions observed within folded RNA molecules (Fig. 4E) (17). Collectively, these interactions enable the holoenzyme to coordinate and selectively identify the shape of a DNA helix.

This shape-selective recognition strategy of the DNA stem is complemented by sequence-specific interactions between the holoenzyme and single-stranded regions of the DNA target. Phylogenetically covarying base-pairs are formed between substrate-recognition regions of the intron and single-stranded DNA nucleobases downstream of the DNA stem (35). In the holoenzyme, we not only identify these critical WC pairings, but we also observe an unexpectedly complex network of interactions mediated by the spacer DNA that connects the stem with the IBS sequences. This sequential network of DNA IBS elements and the adjacent spacer interactions begins with the nucleotide located immediately downstream of the insertion site (dT36), which forms a single-base-pair interaction (EBS3-IBS3) with a nucleotide extending from the D1d coordination loop within the intron (A231) (Fig. 4F). Stacked atop this pair is a short helix formed via base-pairings between the subsequent stretch of DNA nucleotides (IBS1: dT35, dT34, dT33 and dC32) and a second substrate recognition loop that projects from the terminus of intron D1d (EBS1: A184, A185, A186 and G187) (Fig. 4F). Like the short codon-anticodon helix in the ribosome (36), the EBS-IBS1 duplex is further stabilized via formation of an A-minor motif between A75 and the dC32-G187 base-pair (Fig. 4G). Intriguingly, the structure reveals that EBS1-IBS1 is not limited to four contiguous base-pairs, rather, it is extended by an additional base-pair that is formed between the next consecutive nucleotide (A188) in the EBS1 loop and a discontinuous nucleotide from the DNA spacer region (dT30). Indeed, the intervening DNA nucleotide (dT31) is extrahelical and stabilized by interactions with protein residues (vide infra) (Fig. 4H). Through these sequential stacking networks, supported by contacts with the protein (i.e., dT36O4 with K361), the intron achieves stable, base-pairing specificity with the DNA target.

Nucleotides within the DNA spacer region participate in binding the RNP, adopting an ordered structure that engages in specific interactions with the holoenzyme. Rather than forming a helical stack, the spacer nucleotides (dA28, dT29, dT30 and dT31) form an unusual motif in which the nucleotides splay in alternating directions on either side of the central phosphate spine (Pauling-like DNA), thereby exposing a large interaction interface to the adjacent DBD (Fig. 4, F and H). Amino acids from the DBD intercalate between the DNA spacer nucleotides while forming an abundance of interactions with both the bases and the phosphate backbone (Fig. 4H). For example, N3 of dT31 interacts with amide oxygens of N378 while its adjacent phosphate oxygens interact with proximal arginine residues (R381 and R382). Together, these interactions stabilize an unusual backbone conformation that enables the dT30-A188 pair to form atop the EBS-IBS1 helix. In turn, these interactions with the DBD pull the DNA into place, positioning the specialized barb-like structure formed by the α-helical bundle within the DBD at the base of the DNA stem (Fig. 4, B and H).

### Retroelement Primed for Attack

**Figure 5.**
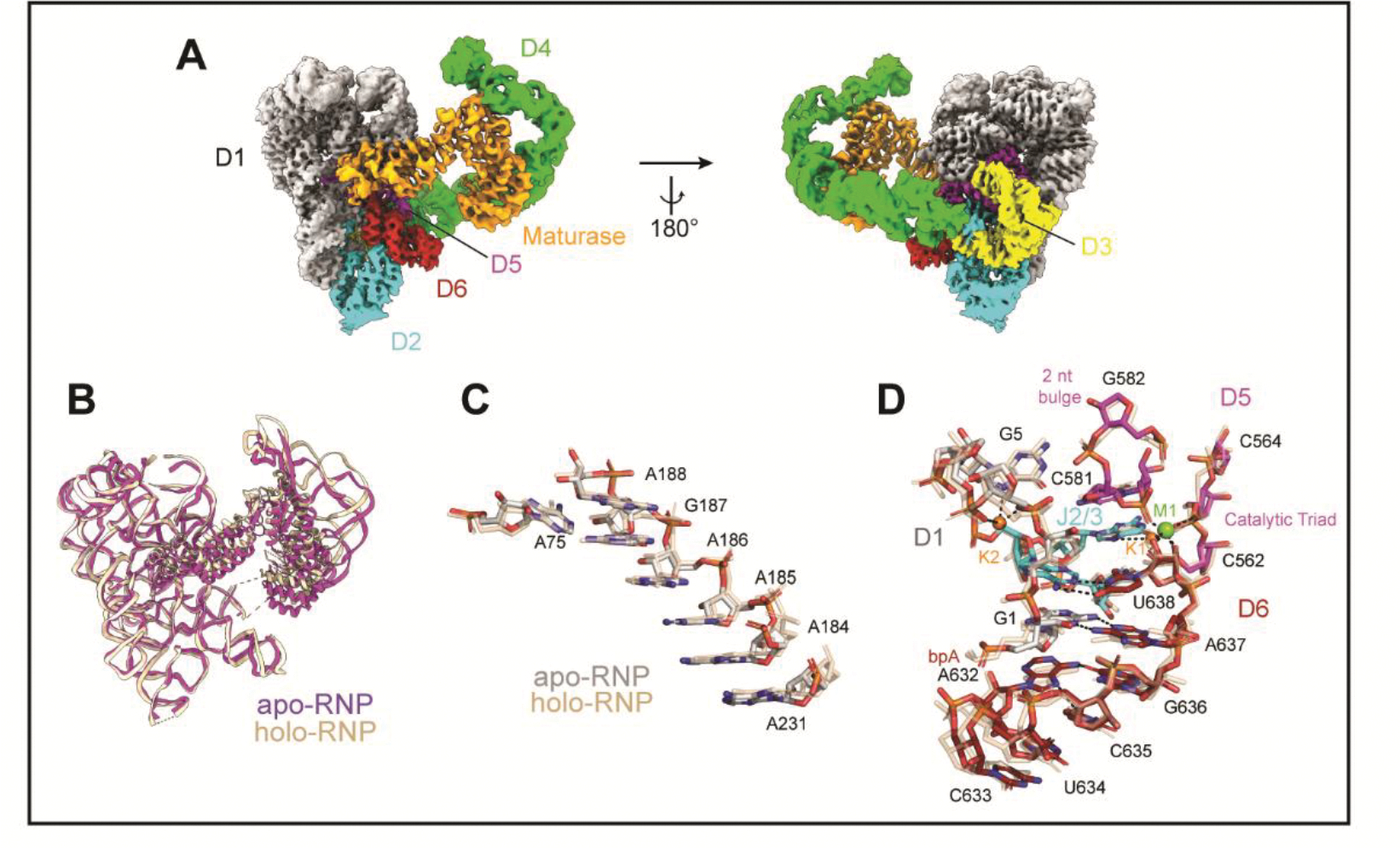
A Retroelement Poised to Attack **(A).** Composite cryoEM map of the apo-RNP. **(B-D).** Comparison of **(B)** the backbone traces, **(C)** the position of the EBS1 recognition sequences and **(D)** the active site of the apoRNP (colored) and holoRNP (wheat).

To better understand molecular rearrangements that might occur when the intron retroelement binds to DNA substrate, we solved the structure of the apo-RNP, visualizing the free intron-maturase complex at a resolution of 3.6Å (Fig. 5A and fig. S9). We were surprised to observe that the apo-RNP has an architecture that is almost identical to that of the complex bound to DNA, and that substrate binding induces only minor changes in the structure. Most remarkably, the RNP active site remains completely intact (Fig. 5, A and B) (9–11). The maturase does not change its orientation in the absence of DNA, remaining coordinated at two anchor points along the D4a arm, with the thumb and DBD inserted into the active site to participate in catalysis (Fig. 5A). In this configuration, the binding interface for the target DNA is maintained, enabling the RNP to readily recognize an incoming DNA target and rapidly engage in retrotransposition (Fig. 5A). Upon recognition of the DNA stem-loop, the RNP (palm, fingers and D4a) appears to become more rigid, as we observe a concomitant increase in local resolution at these positions (fig. S3B and S9B and movie S5 and S6). This is reminiscent of many protein enzymes, whereby docking of ligand into the active site freezes out local motions and locks the substrate in place. In previous ligand-free intron structures, EBS nucleotides were found to be disordered or rearranged (23, 24, 26). Here, we observe that the positions of the EBS nucleotides are unchanged, likely due to the presence of the maturase. These findings reinforce the mechanistic role of the protein as a stabilizing catalytic component (Fig. 5C). Further evidence of a pre-formed catalytic core includes the persistence of the heteronuclear metal ion cluster, which remains organized around the lariat, although M2 is not visible in this case (Fig. 5D).

## Discussion

### Insights into Protein Facilitated Ribozyme Catalysis

The structures presented here reveal how components of the holoenzyme help promote activity of the ribozyme core. The protein buttresses the catalytic residues responsible for specifically positioning the DNA substrate, providing a missing link in understanding how maturases unlock the full catalytic potential of group II intron ribozymes (Fig. 2). Interactions with protein residues stabilize the intron substrate recognition loops and precisely arrange nucleotides for DNA binding. These interactions contribute to proper orientation of reaction components throughout ribozyme catalysis. Our findings provide a direct mechanistic role for the protein cofactor, and they help explain the lowered salt and magnesium requirements in its presence (38).

Prior studies, due to resolution limitations or construct design, were unable to identify a specific function for the maturase protein, except in transient D6 stabilization (9). By contrast, we can now show that not only are the protein thumb and DBD proximal to D1 catalytic residues, but also that they have critical roles in stabilizing substrate binding. Given its analogous spatial placement, it is possible that Prp8 may play a similar role during spliceosomal catalysis (39) (fig. S10).

### RNP Recognition of DNA Structure: An Expanded Recognition Repertoire

The high-resolution cryoEM structures we provide here offer an unprecedented glimpse into RNP strategies for recognizing DNA (Fig. 1B). The holoenzyme structure reveals a stem-loop DNA nestled within the retroelement, bound to RNA and protein. Within this cleft, the protein assists in positioning the insertion site and aligning the DNA stem for steric fit against the complementary maturase surface (Fig. 3). Additional aspects of the unusual recognition strategy include splayed Pauling-like DNA, a stabilizing A-minor motif, and intermolecular stacking moieties involving both RNA and protein (Fig. 4). These interactions highlight the symbiotic nature of RNA and protein and underscore the multiplicity of strategies available to RNPs for achieving selective substrate recognition. Proteins have long been known to recognize DNA structures, but here we show that RNPs, with their expanded repertoire of DNA molecular recognition determinants, have this ability too (40).

The DNA stem loop motif is exclusive to IIC introns, which contain an abbreviated D1 scaffold and short exon recognition sequences (7). Intriguingly, in the more highly evolved IIA and IIB introns, the RNP binding motif that we find occupied by the DNA stem in IIC introns is instead replaced by intron insertion motif D1d2, an RNA subdomain that includes EBS2, which is absent in the IIC class (7). Comparison of this region across intron classes suggests that EBS2 evolved to imitate the target DNA stem (fig. S11). Indeed, the DNA stem motif structurally resembles the EBS2-IBS2 interactions typical of IIA and IIB introns and it functionally emulates EBS2 by anchoring the DNA substrate to the RNP. This mimicry suggests that initial recognition of a structured DNA motif by the more primitive IIC introns was replaced by novel RNA domains within the intron itself, resulting in longer target recognition sequences that provided greater base-pairing specificity for the retroelement.

### Implications for Reverse Splicing and Reverse Transcription

A surprising feature of our structures is the way that the protein is positioned within the holoenzyme. Encircled by RNA, the exterior surfaces of the protein are occupied, but the concave interior of the protein, adjacent to the catalytic core, is conspicuously solvent accessible, which has functional implications. During reverse splicing, the D6 helix undergoes a conformational change that places the lariat linkage into the active site (9). To accomplish this, D6 disengages from D2 and swings 90° upward, contacting D1c and a basic patch on the protein thumb. Our structures do not preclude D6 helix dynamics, as there is ample space for a similar movement and the regions that D6 contacts are accessible. The open architecture we observe provides a direct route for DNA to approach the RT active site, as it remains unobstructed by other intron domains and can readily accommodate an entire hybrid duplex for reverse transcription (41). This suggests that initiation of RT activity, within the current holoenzyme assembly, may be possible without significant conformational rearrangement.

### Retroelement Poised to Attack

Group II intron retroelements are proliferative, invasive agents and our structures explain why. The apo-retroelement is completely poised to react and does not require any reorganization of structure upon target DNA binding. The arrangement of the active site, from substrate recognition nucleotides to the heteronuclear metal ion cluster to the DNA binding interface, is preserved despite the absence of DNA substrate (Fig. 5). This prearranged organization is consistent with the biological role of group II introns as parasitic genetic elements (42). Use of the same catalytic core from splicing to integration eschews the need for major rearrangements or host cofactors and allows complete autonomy, which is highly advantageous for a genetic parasite.

Total integration of the RNP requires faithful and accurate reverse transcription of the intron sequence, including the long ORF that encodes the protein, after insertion. This is accomplished using the RT activity of the multifunctional IEP. MarathonRT, the protein within the holoenzyme visualized here, is a well characterized, robust, accurate and ultraprocessive RT enzyme capable of copying through long, structurally complex templates (43). The intimate association of the parent intron with this protein allows access to its exceptional reverse transcriptase properties and ensures that the intron sequence, which is pivotal to its tertiary architecture, is preserved, allowing the retroelement to continually propagate.

### Implications for Modern Retroelements

Study of group II intron complexes provides a window into our understanding of non-LTR retrotransposons, such as the L1 RNP, an active mobile element that continues to disperse in human genomes (12, 13). Computationally predicted structures of ORF2p, the mobility factor of L1, show that its RT and thumb domain resemble that of the maturase, MarathonRT (fig. S12) (44). ORF2p contains an additional N-terminal endonuclease and a C-terminal extension, but these domains do not block the exterior basic surfaces of the RT and thumb. MarathonRT and ORF2p are evolutionarily related, and they are implicated in similar mobility mechanisms, so it is possible that the same surfaces are used for anchoring and substrate recognition (45). Given the lack of structural information on L1 and the strong parallels between systems, our work provides a starting point for imagining how a similar retroelement like L1 might assemble.

In conclusion, we present high-resolution cryoEM structures that reveal the detailed molecular architecture of an intron retroelement complex, thereby shedding light on the mechanism of maturase-facilitated ribozyme catalysis and retrotransposition. The structures reveal novel strategies for RNP interaction with DNA structural motifs and they explain the proliferative nature of group II intron retrotransposons, with implications for understanding the spliceosome and evolutionarily related retroelements. This study underscores the multiplicity of molecular interactions and strategies that are employed as diverse biomolecules carry out the reactions central to genomic stability and plasticity.

## Supplementary Material

Movie_S1

Movie_S2

Movie_S3

Movie_S4

Movie_S5

Movie_S6

Supp_Material

## Data Availability

All data are available in the main text and the supplementary materials. Cryo-EM maps are available in the Electron Microscopy Data Bank with codes EMD-26550 (holoRNP) and EMD-26549 (apoRNP). Structural models are available in the Protein Data Bank with PDB accession codes 7UIN (holoRNP) and 7UIM (apoRNP).
