# Supplementary material for: Structures of a Mobile Intron Retroelement Poised to Attack Its Structured DNA Target
Source: Science. Author manuscript; Available in PMC 2023 May 17. (PMC10190682; doi:10.1126/science.abq2844)
Supplement: Supp_Material [file NIHMS1870836-supplement-Supp_Material.docx]

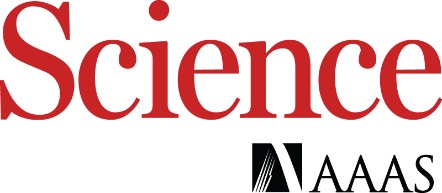


Supplementary Materials for

CryoEM Structures of a Mobile Intron Retroelement Poised to Attack Its Structured DNA Target

**Authors:** Kevin Chung^1^†, Ling Xu^2, 3^†, Pengxin Chai^1^, Junhui Peng^4^, Swapnil C. Devarkar^1^, Anna Marie Pyle^2, 3*^

Correspondence to: Anna Marie Pyle, anna.pyle@yale.edu

**This PDF file includes:**

Materials and Methods

Figs. S1 to S12

Table S1

References (*46-65*)

**Other Supplementary Material for this manuscript includes the following:**

Movies S1 to S6

MDAR Reproducibility Checklist

MATERIALS AND METHODS

**Protein Purification**

The full-length protein (MarathonRT) was purified as previously described with minor changes (*15*). The recombinant protein was cloned into pET-SUMO vector (ThermoFisher) and was directly fused to the C-terminus of a 6xHis-SUMO tag. The plasmid was transformed into Rosetta II (DE3) *Escherichia coli* cells (Millipore). The cells were grown at 37°C in LB medium supplemented with 50 µg/mL kanamycin and 17 µg/mL chloramphenicol, first in a small 200 mL culture overnight, before transferring to a large 2L culture, which was grown to an A_600_ of 0.8 to 1.0. Protein expression was induced at 16°C overnight by adding 0.5 mM of isopropyl-β-d-thiogalactopyranoside (IPTG). Cells were harvested and resuspended in lysis buffer (25 mM NaHEPES, pH 7.5, 1 M NaCl, 10% glycerol and 2 mM β-mercaptoethanol (βME)) containing dissolved protease inhibitor (Sigma). Cells were lysed by passing the resuspension through a microfluidizer and the lysate was centrifuged at 13K rpm to remove precipitates. The supernatant was loaded onto nickel-chelating columns (GE Healthcare) for purification. The column was washed with lysis buffer and then wash buffer (25 mM NaHEPES, pH 7.5, 150 mM NaCl, 10% glycerol, 2 mM βME, and 25 mM imidazole) and eluted with elution buffer (25 mM NaHEPES, pH 7.5, 150 mM NaCl, 10% glycerol, 2 mM βME, and 300 mM imidazole). The eluted protein was then incubated with ULP1 SUMO protease at 4°C for 1 h to cleave the N-terminal 6xHis-SUMO tag. After tag cleavage, the protein was loaded onto a 5 mL HiTrap HP column (GE Healthcare), equilibrated with buffer A (25 mM K-HEPES, pH 7.5, 150 mM KCl, 10% glycerol and 1 mM DTT). The protein was eluted by running a gradient to buffer B (25 mM K-HEPES, pH 7.5, 2 M KCl, 10% glycerol and 1 mM DTT). The peak fractions were pooled, concentrated to 5 mL and injected onto a Superdex S200 gel filtration column (GE Healthcare) equilibrated with buffer A. After gel filtration, the peak fractions from the S200 column were pooled, concentrated to 50 mg/mL, flash frozen under liquid nitrogen and stored at −80°C.

**RNA Transcription and Purification**

The DNA sequence of the full-length group II intron was designed to include the T7 RNA polymerase promoter, the RNA coding sequence (with maturase ORF deleted), flanking exons, and a restriction site for BamHI in pBluescript (Invitrogen). The plasmid template was linearized using BamHI (Invitrogen) and then purified by ethanol precipitation. RNA samples were synthesized by *in vitro* transcription with purified T7 RNA polymerase in 1 mL reactions. The reaction mixture contained 10 mM Tris pH 7.5, 1 mM spermidine, 0.01% (v/v) Triton X-100, 10mM DTT, 5 mM NTPs, 15 mM MgCl_2_, 30 μg linearized DNA template, and 1 μM T7 RNA polymerase. The reaction was performed at 37°C for 4h. After transcription, the reaction mixture was treated with 0.1 volumes of 0.5 M EDTA and separated on a 5% denaturing polyacrylamide gel after adding denaturing loading dye. The RNA band was eluted with a 10 mM MOPS pH 6.0 buffer at 4°C overnight and then filtered with a 0.2 μm vacuum filter (ThermoFisher). The RNA sample was further purified by adding 0.1V 5M NaCl, 3V absolute alcohol and incubated at -80°C overnight. Then, the transcription products were centrifuged, the supernatant was discarded, and the precipitate was air dried before being dissolved in 10 mM MOPS, pH 6.0.

**Lariat-Maturase Complex Formation and Purification**

To obtain lariat-maturase RNP complex, a forward splicing reaction was conducted in 0.5 mL reactions containing purified intron RNA, purified intron maturase, 50 mM NH_4_-MES pH 6.0, 30 mM MgCl_2_, 500 mM (NH_4_)_2_SO_4_, 1 mM DTT, and RNase Inhibitor (ThermoFisher). The purified intron RNA was first mixed with buffer and water and allowed to fold by heating to 95°C for 2 minutes followed by cooling at 25°C for another 2 minutes. Next, MgCl_2_, (NH_4_)_2_SO_4_, DTT, and purified maturase were added. The reaction was incubated at 42°C for 1 hour after which it was centrifuged at 13K rpm for 2 minutes to remove the precipitants. The supernatant was loaded onto a HiLoad 10/300 S200 Increase column (GE Healthcare) equilibrated with buffer containing 50 mM NH_4_-MES pH 6.0, 30 mM MgCl_2_, 200 mM (NH_4_)_2_SO_4_, and 1 mM DTT and separated. Fractions were run on a 5% denaturing gel and on a 5% native gel to check the contents. Those corresponding to the lariat-maturase complex were pooled and used in subsequent assays (see below).

**Size Exclusion Chromatography-Multi Angle Light Scattering (SEC-MALS)**

SEC-MALS was performed on samples (500 µL) using a Superdex 200 10/300 HR SEC column (GE Healthcare) connected to a High-Performance Liquid Chromatography System (HPLC) Agilent 1200 (Agilent). The elution from SEC was monitored using a DAWN Heleos-II spectrometer (Wyatt Technology) coupled to an Optilab T-rEX (Wyatt Technologies) interferometric refractometer. The SEC-UV/LS/RI system was equilibrated in a buffer containing 50 mM NH_4_-MES pH 6.0, 30 mM MgCl_2_, 200 mM (NH_4_)_2_SO_4_, and 1 mM DTT. Chemstation software (Agilent) controlled the HPLC operation and data collection from the multi-wavelength UV/VIS detector, while the ASTRA software (Wyatt) collected data from the refractive index detector, the light scattering detectors, and recorded the UV trace at 295 nm. The molecular weight (MW) was determined across the entire elution profile in intervals of 1 sec from static LS measurement using ASTRA software.

**Analytical Ultracentrifugation**

Sedimentation velocity analytical ultracentrifugation (SV-AUC) experiments were performed using a Beckman XL-A centrifuge with an An-60 Ti rotor (Beckman Coulter) at the Yale Chemical and Biophysical Instrumentation Center (CBIC) as in previous protocols (*24*). The lariat and lariat-maturase complex were in buffers containing 50 mM NH_4_-MES pH 6.0, 30 mM MgCl_2_, 200 mM (NH_4_)_2_SO_4_, and 1 mM DTT. Purified protein was in 25 mM K-HEPES, pH 7.5, 150 mM KCl, 10% glycerol and 1 mM DTT. Sample concentrations were adjusted to an absorption of 0.5 at 260 nm for RNA containing samples and at 280 nm for protein samples. The samples were allowed to equilibrate at 20°C for 60 min in the instrument before collecting 150 radial scans in duplicate at 50,000 rpm. The entire data collection process took place over 16 hours. Data were analyzed using a continuous c(s) distribution model as implemented in Sedfit. Buffer density and viscosity values used were 1.0381 g/mL and 0.0111031 poise respectively.

**Intron-Retroelement Complex Purification**

To purify the ternary retroelement complex, we performed a pulldown experiment with tagged DNA. The DNA (IDT) target sequence contains a 5’ desthiobiotin tag and has a sequence as follows: GAGAGCAGGGGCTATGAACCTGCTCTCATTTCTTTTG. DNA was folded by heating to 95°C for 2 minutes and cooling at 25°C for 2 minutes. 2.5 μg of annealed DNA was added to a 0.5 mL solution of purified lariat-maturase RNP and allowed to bind at 25°C for 30 minutes. Next, 100 μL of pre-washed softlink avidin resin (Promega) was added and the mixture was incubated at 25°C for 30 minutes. The mixture was spun down and the supernatant was discarded. The resin was washed three times with 0.5 mL of buffer (50 mM NH_4_-MES pH 6.0, 30 mM MgCl_2_, 200 mM (NH_4_)_2_SO_4_, and 1 mM DTT). The retroelement complex was eluted with elution buffer (50 mM NH_4_-MES pH 6.0, 30 mM MgCl_2_, 200 mM (NH_4_)_2_SO_4_, 1 mM DTT, and 5 mM biotin) by rotating at 25°C for 30 minutes. The eluant containing the assembled lariat-maturase-DNA complex was used for further structural studies.

***In vitro* Reverse Splicing Assays**

Purified lariat-maturase RNP complex was mixed with radiolabeled DNA to perform retrotransposition assays. Briefly, 5’ end labelling was done by mixing [γ-^32^P] ATP, PNK Buffer (NEB), T4 Kinase (NEB), and DNA. The mixture was incubated at 37°C for 30 minutes and the kinase enzyme was deactivated by heating at 65°C for 30 minutes. DNA was desalted by passing through a G25 column (ThermoFisher) by spinning at 700 g for 2 minutes. 5’ end labeled primers were then purified by ethanol precipitation, spun down, air dried and resuspended in water. For the intron insertion reaction, radiolabeled primer DNA was folded by heating at 95°C for 2 minutes and cooled at 25°C for 2 minutes. The DNA was mixed with purified lariat-maturase RNP complex in 50 mM K-HEPES pH 7.5, 4 mM MgCl_2,_ and 50 mM KCl and allowed to react at 42°C. Aliquots were taken from the mixture at various time points and quenched by combining with formamide dye containing xylene cyanol and bromophenol blue. Samples were then analyzed on a 15% denaturing gel. Gels were imaged using a Typhoon phosphorimager.

**Grid Preparation and Data Collection**

For cryoEM analysis of the group II intron retroelement, 4 μl of the purified lariat-maturase complex (apo-RNP) or the assembled lariat-maturase-DNA complex (holo-RNP) was loaded onto plasma cleaned QuantiFoil Cu R1.2/1.3 300-mesh grids (Quantifoil) prepped with an extra layer of carbon. Using a condition of 100% humidity and 4°C, the grids were blotted before being plunged into liquid ethane and frozen in liquid nitrogen. SerialEM was used for data collection. Micrographs were recorded in a Titan Krios microscope (FEI) operating at 300 kV, equipped with a K3 Summit direct electron detector (Gatan) operating in counting mode. A tilt-specimen data collection was employed to address the preferred sample orientation issue. For holo-RNP 10312, 1321 and 2851 micrographs were collected at 0°, 10°, and 30° using two different grids, one for the non-tilted data and one for the tilted dataset. For apo-RNP, 3006, 609, 657, 3184, and 549 micrographs were collected at 0°, 10°, 20°, 30° and 40° tilt angles respectively. A nominal magnification of 105,000x and a defocus range of -0.5 µm to -2.0 µm and -1.0 µm to -2.5 µm were used for imaging the holo-RNP and apo-RNP respectively, giving an effective pixel size of 0.832Å at the specimen level. For the holo-RNP, each micrograph was dose-fractionated to 40 frames under a dose rate of 17.6 e-/pixel/s, with a total exposure time of 2 s and a frame exposure time of 0.05 s, resulting in a total dose of 50.85 e^-^/A^2^. For the apo-RNP, each micrograph was dose-fractionated to 40 frames under a dose rate of 17.4 e^-^/pixel/s, with a total exposure time of 2 s and a frame exposure time of 0.05 s, resulting in a total dose of 50.27 e^-^/A^2^.

**CryoEM Data Processing**

CryoEM data processing workflows are outlined as indicated in fig. S3A and fig. S9A. Recorded movie frames were processed using cryoSPARC v3.0 (*46, 47*). Patch motion correction and CTF estimation were performed using default parameters in cryoSPARC. Exposures were curated and micrographs with ice contamination, excessive motion or damaged regions were removed. For the holo-RNP ternary complex, template picking was used to select particles from the untilted dataset (10 508 micrographs). For the tilted dataset (3 976 micrographs), 2D classification screened, template picked particles from 100 micrographs were used to train a Topaz (*48*) model for neural network-based picking from the entire tilted dataset. Particles from each dataset were extracted using a box size of 384 pixels, binned twice to 192 pixels, and were separately filtered through several rounds of 2D classification. Final 2D classes from the untilted dataset were manually separated into two subsets that represented (1) the dominant, front views and (2) the other, lesser represented views which had 3 136 194 particles and 702 685 particles, respectively. To improve particle distribution, particles from the dominant views were randomized into 10 subsets, from which one subset of particles was selected for furthering processing. This randomly selected subset of particles was combined with those from classes representing ‘other’ views and the particles from the tilted dataset, resulting in a total of 1 617 845 good particles. An initial reconstruction was performed using all good particles. Next, alignment free 3D classification, using unbinned, re-extracted full box size particles, was performed using a loose mask covering the entire holo-RNP complex. Particles were separated into ten different classes from which one class, containing 300 344 particles, with the best, continuous density was selected. Non-uniform refinement (*47*) of the selected class was done to obtain the full holo-RNP map at 2.8Å. 3D variability analysis (*49*) (movie S5) was performed to identify continuous motions within the holo-RNP ternary complex. Subsequent focused refinement with a specified fulcrum position in the middle of the DNA hairpin was done on the left and right halves of the holo-RNP complex. After CTF refinement, a 2.7Å map for the holo-RNP (left) and 3.0Å map for the holo-RNP (right) were obtained as evaluated using a GSFSC criterion of 0.143.

A similar strategy was used for cryoEM reconstruction of the apo-RNP. 2D classification screened template picked particles from a subset of 100 micrographs were used to train a Topaz (*48*) model first for picking from the smaller subset of micrographs before picking from the full dataset of 7500 micrographs. Picked particles were extracted with a box size of 384 pixels and binned twice to 192 pixels. Particles were filtered by several rounds of 2D classification, resulting in 454 725 good particles. After a consensus refinement with re-extracted unbinned particles, focused classification without alignment was performed on the ribozyme portion of the apoRNP. It was found that there was good density in all classes so we proceeded with focused refinement with a mask on the protein and D4a arm. Two of the eight classes, totaling 136 222 particles, displayed good density for the maturase protein and RNA D4a arm. Particles from these two classes were selected and non-uniform refinement of these particles yielded a 3.6 Å map of the full apo-RNP. 3D variability analysis indicated that there were multiple modes of motion in 1) the basal portion of the D4 arm connecting to the ribozyme core, and in 2) the D6 helix and protein/D4a arm (movie S6). Subsequently, focused refinement with a mask on the protein and D4a portions of the apo-complex was performed with a fulcrum placed at the C-terminus of the maturase protein.

After CTF refinement, this allowed us to obtain a 3.9 Å map of the protein and D4a arm of the apo-RNP complex. As the ribozyme core remains static relative to the protein and D4a arm as confirmed by focused classification, focused refinement and CTF refinement on just this region was conducted, generating a 3.0 Å reconstruction of the ribozyme portion of the apo-RNP complex as evaluated by the GSFSC threshold of 0.143. Directional anisotropy analysis for the three maps of the holo-RNP complex and apo-RNP were performed using 3DFSC (*50*). Composite maps were manually generated for the holo-RNP and apo-RNP using focused refined maps aligned to the consensus map.

**Model Building and Refinement**

Model building was initiated by docking an intron crystal structure (PDB: 3EOH) and an AlphaFold generated model of the maturase (MarathonRT) into the overall cryo-EM density map of holo-RNP using UCSF Chimera (*44, 51, 52*). The holo-RNP model was then manually rebuilt in COOT into the focused refined maps, aligned to the overall holoRNP complex map, to accommodate for sequence and structural changes, and novel segments (*53*). The DNA in the model was built de novo in COOT. dT13 does not have clear density and appears to be extrahelical in our model. There is some density at the 5’ end of the DNA substrate that was not modeled, which presumably is from the desthiobiotin tag. For the D4a arm, a stretch of high-resolution density along the backside of the maturase palm domain allowed assignment of RNA sequence based on the cryoEM densities and identification of purine and pyrimidine nucleobases. This allowed the D4a arm to be extended bidirectionally to the distal loop and towards the basal portion that connects to the ribozyme core. The three-way junction from the base of the D4 arm towards the D4a and D4b arm extensions exhibited lower density and were not modeled. Density for the distal portion of D6 was weak, but this region was modelled in as a helix based on data from lower resolution classifications and 3D variability analysis that show that this domain forms a canonical but dynamic helix. The α-α’ kissing loop, while visible in the cryoEM density, is dynamic as seen in 3D variability analysis and was not modeled. The pentaloop of D2a was not visible in the cryoEM reconstructions. The maturase was modeled in save for an alpha-loop and beta-hairpin in the finger domain of the protein. All other portions of the RNA and protein were modeled in.

The generated holo-RNP model was docked into the apo-RNP maps, which was manually and computationally refined (*54*). The final holo-RNP and apo-RNP model were improved by iterative rounds of real-space refinement against the full consensus map for the holoRNP and the composite map for the apoRNP in PHENIX (*55*) using secondary structure restraints for both RNA, protein and DNA, as well as Ramachandran and rotamer restraints for protein chains, and subsequent rebuilding in COOT (*55-57*). Model building and validation statistics are listed in Table S1. Molecular interfaces and interaction networks were analyzed for each of the models using PDBePISA (*58*). Figures were prepared with Pymol (<https://pymol.org/2/>) and Chimera/ChimeraX (*52, 59*) .

**Analysis of DNA Stem-Loop Motif**

Group IIC sequences were collected from the Bacterial Group II Intron Database (*14*). Sequences 5’ of the DNA insertion site were used for secondary structure prediction by RNAfold (*60*). As the DNA stem loop motif is located proximal to the exon junction, only the last 50 nt of the 5’ flanking sequences were used. From this trimmed set of sequences, a further 5 nt were removed from the 3’ end to account for IBS1 nucleotides, which are known to bind the intron EBS1 sequence and would not be involved in the DNA stem-loop secondary structure. The stem and loop length for each input sequence was recorded, and the averages were calculated. Helix parameters for the DNA stem including major and minor groove width, and helical twist were calculated using 3DNA (*61*).

**Protein Conservation**

Group IIC maturase protein sequences were collected from the Bacterial Group II Intron Database (*14*). In total 91 sequences of functional and active maturase proteins were selected and used for alignment with ClustalOmega (*62*). Alignments were analyzed and visualized using JalView (*63*).

**
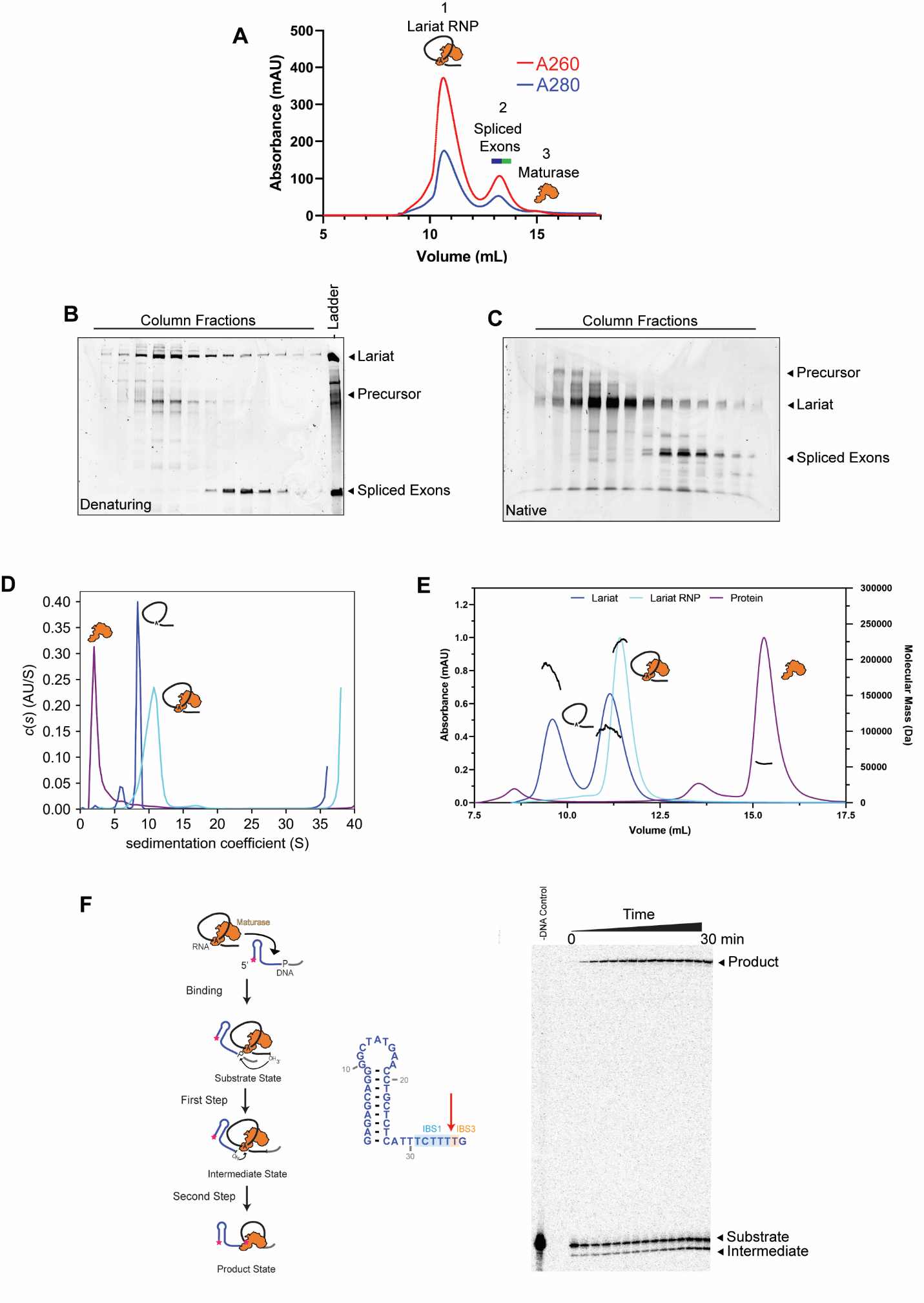
**

**Fig. S1. Purification of a Group II Intron RNP**

**(A).** Size exclusion chromatogram of a group II intron forward splicing reaction. The three peaks are (1) lariat RNP (apo) complex, (2) spliced exons and (3) excess maturase. A_260_ and A_280_ traces are shown in red and blue respectively. **(B).** Denaturing gel of the column purification fractions from (A) with a splicing ladder reference. The three sets of bands correspond to the lariat, unreacted precursor, and spliced exons. **(C).** Native gel of the column purification fractions from (A). The two dominant sets of bands correspond to lariat RNP and spliced exons. **(D).** Sedimentation profiles obtained from analytical ultracentrifugation. The three peaks from lowest to highest sedimentation coefficient correspond to the maturase protein, purified lariat alone, and purified lariat RNP, as in (A). **(E).** SEC-MALS chromatogram of purified lariat, purified lariat RNP and maturase. The purified lariat migrates as two separate species of 195 kDa and 110 kDa (degradation product). The purified lariat RNP appears as a monodisperse peak with a molecular weight of 225 kDa. The maturase protein elutes last and has a measured mass of 54 kDa. **(F).** Cartoon of the reverse splicing reaction (left) - the group II intron integrates into a DNA target. The lariat-maturase RNP binds and recognizes the DNA substrate before inserting into the exon junction followed by opening of the lariat. The red star indicates the 5’ radiolabel used to visualize the substrate (37-nt), intermediate (35-nt) and product (675-nt) states of the reaction. Denaturing gel (right) of the reverse splicing reaction time course with WT DNA substrate (middle).

**
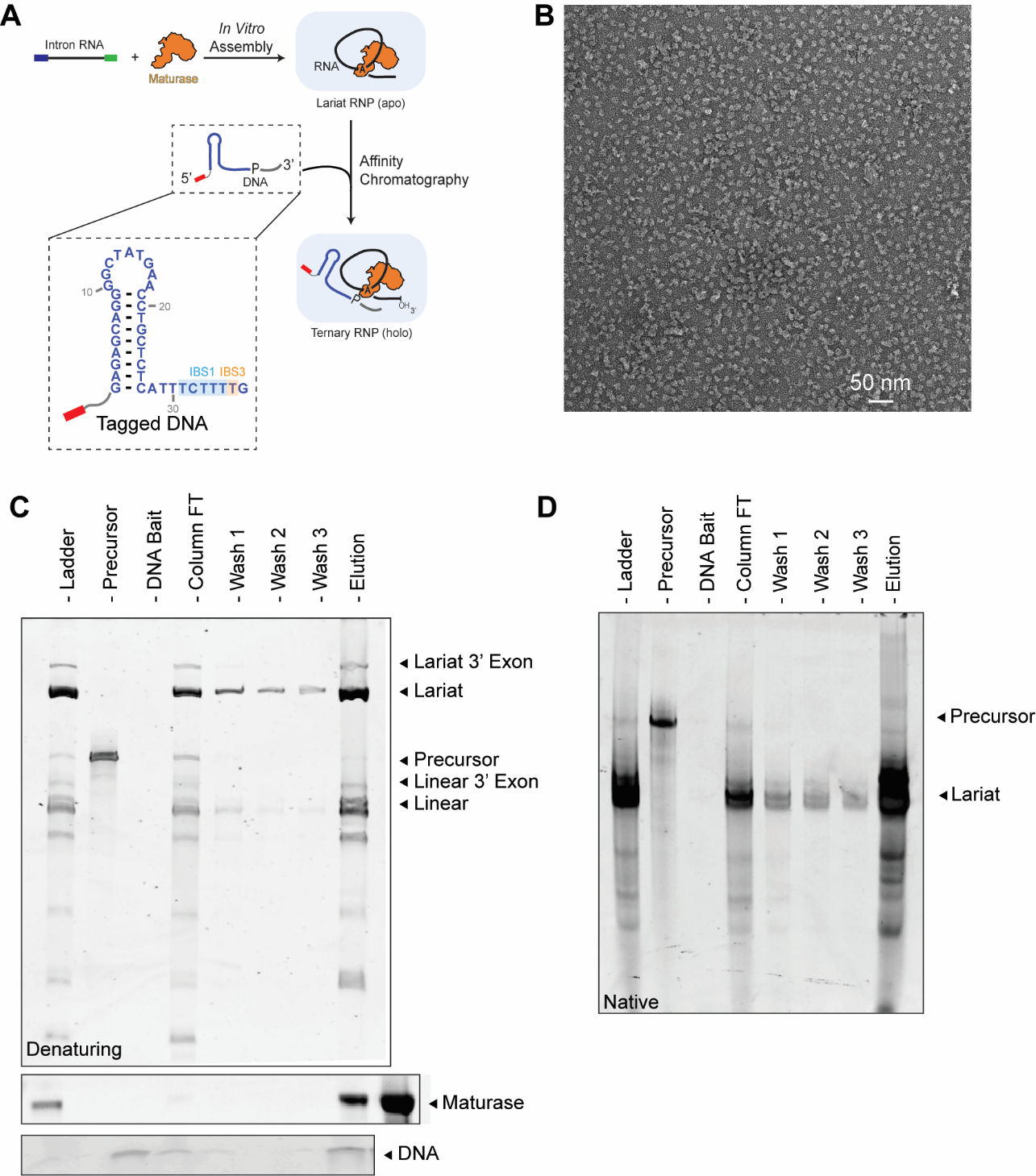
**

**Fig. S2. Purification of a Group II Intron Retroelement**

**(A).** Cartoon of the holo-RNP purification process. Precursor intron RNA was incubated with maturase and reacted to form the lariat RNP complex. The *in vitro* assembled complex was purified and incubated with a tagged, structured DNA target. The secondary structure of the desthiobiotin tagged DNA is shown. Ternary complexes containing lariat RNA, maturase and DNA hairpin were isolated by affinity chromatography by binding the ternary complex to an avidin column and subsequently eluting with biotin. **(B).** Negative stain electron micrograph of the elution fraction from affinity purification. **(C).** Denaturing gel of the affinity chromatography fractions. The middle inset shows a SYPRO Ruby stained protein gel of the same fractions with an additional maturase control. The bottom inset shows a higher percentage denaturing gel of the same fractions. **(D).** Native gel of the affinity purification fractions.

**
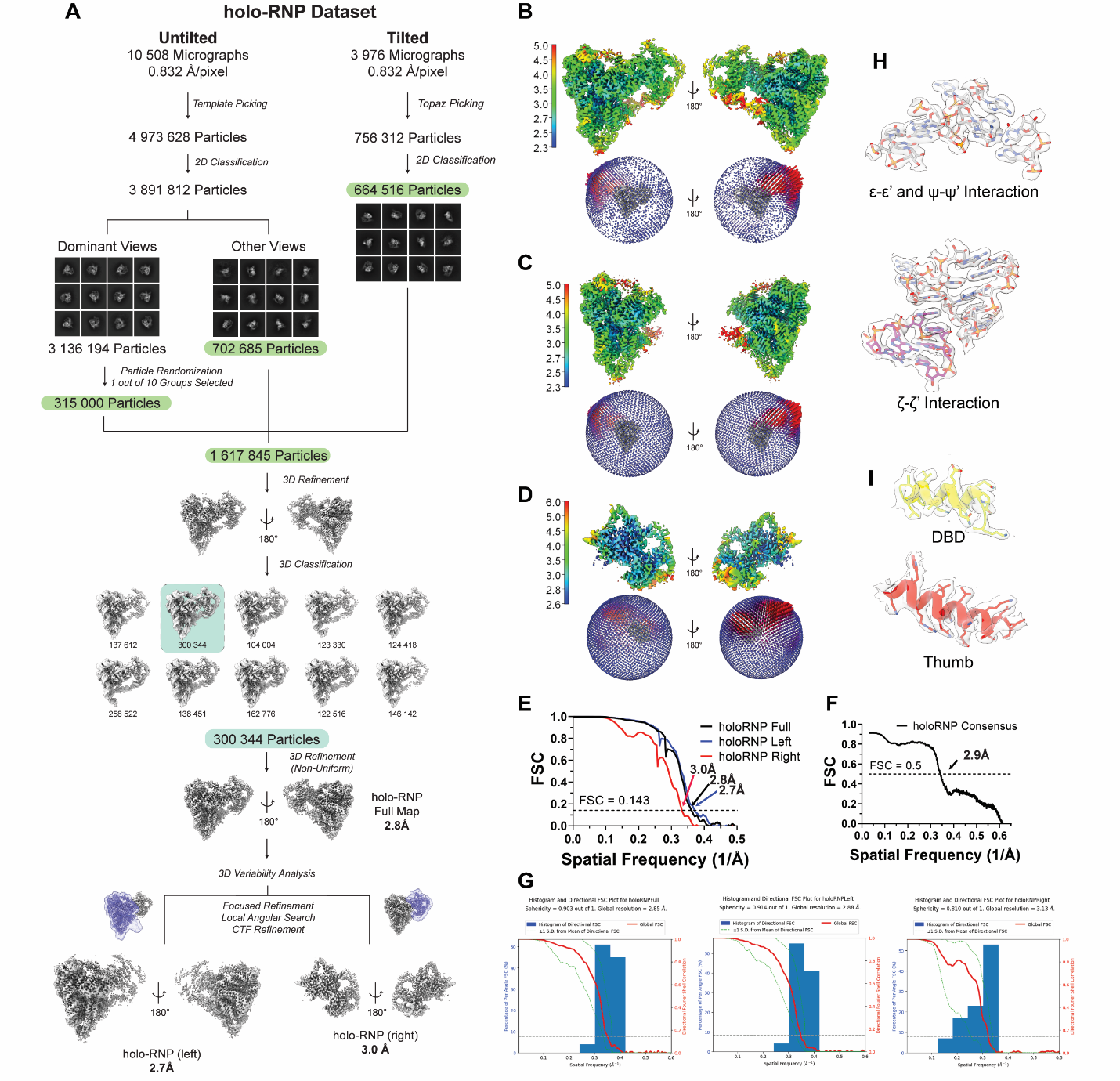
**

**Fig. S3. CryoEM workflow of the ternary lariat-maturase-DNA holo-RNP complex**

**(A).** CryoEM data processing workflow of the lariat-maturase-DNA ternary complex (details in methods) **(B-D).** Local resolution map and particle distribution of **(B)** the holo-RNP full map, **(C)**. the holo-RNP (left) map and **(D)** the holo-RNP (right) map. **(E).** FSC curves with the gold standard threshold of 0.143 for all three holo-RNP complex maps. **(F).** FSC curve for the model refined against the holo-RNP full consensus map. **(G).** 3D FSC analysis of the holo-RNP (full) reconstruction, and the focused refined holo-RNP (left) reconstruction and the holo-RNP (right) reconstruction. **(H-I).** Representative cryoEM densities of **(H)** RNA and **(I)** protein for the holo-RNP ternary complex.

**
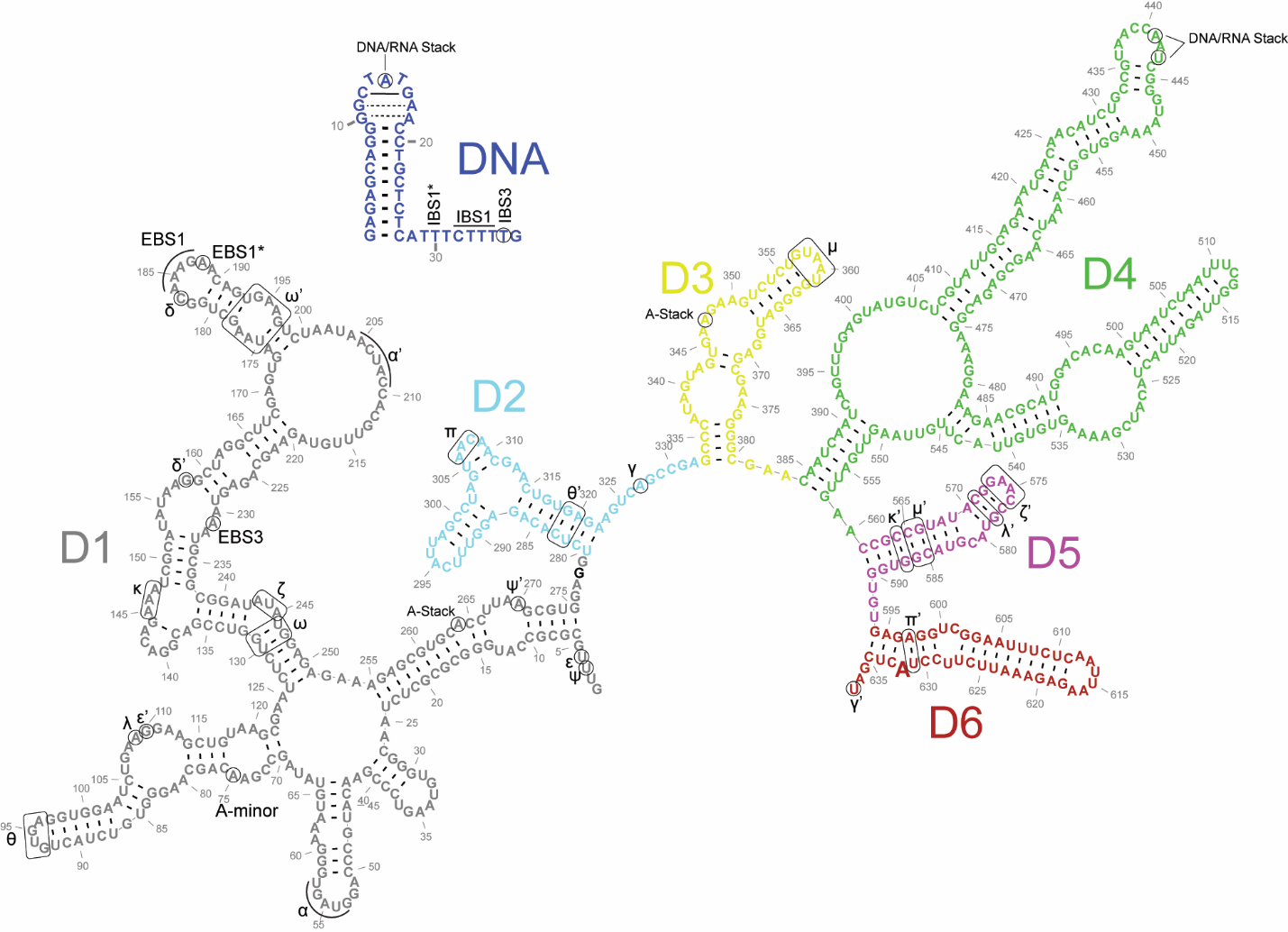
**

**Fig. S4. Secondary Structure Map of the *E.r* Intron and its DNA Target**

Secondary structure map (prepared with RNA2Drawer (*64*)) of the *E.r.* IIC intron and the terminator hairpin DNA target. RNA domains are labeled D1-D6. Tertiary interactions are labeled with Greek letters. Exon binding sequences and intron binding sequences are labeled EBS and IBS, respectively. The average stem and loop lengths for IIC targets were calculated to be 11.5 bp and 4.6 nt respectively. The slightly bulged Ts in the DNA loop indicate extrahelical bases.

**
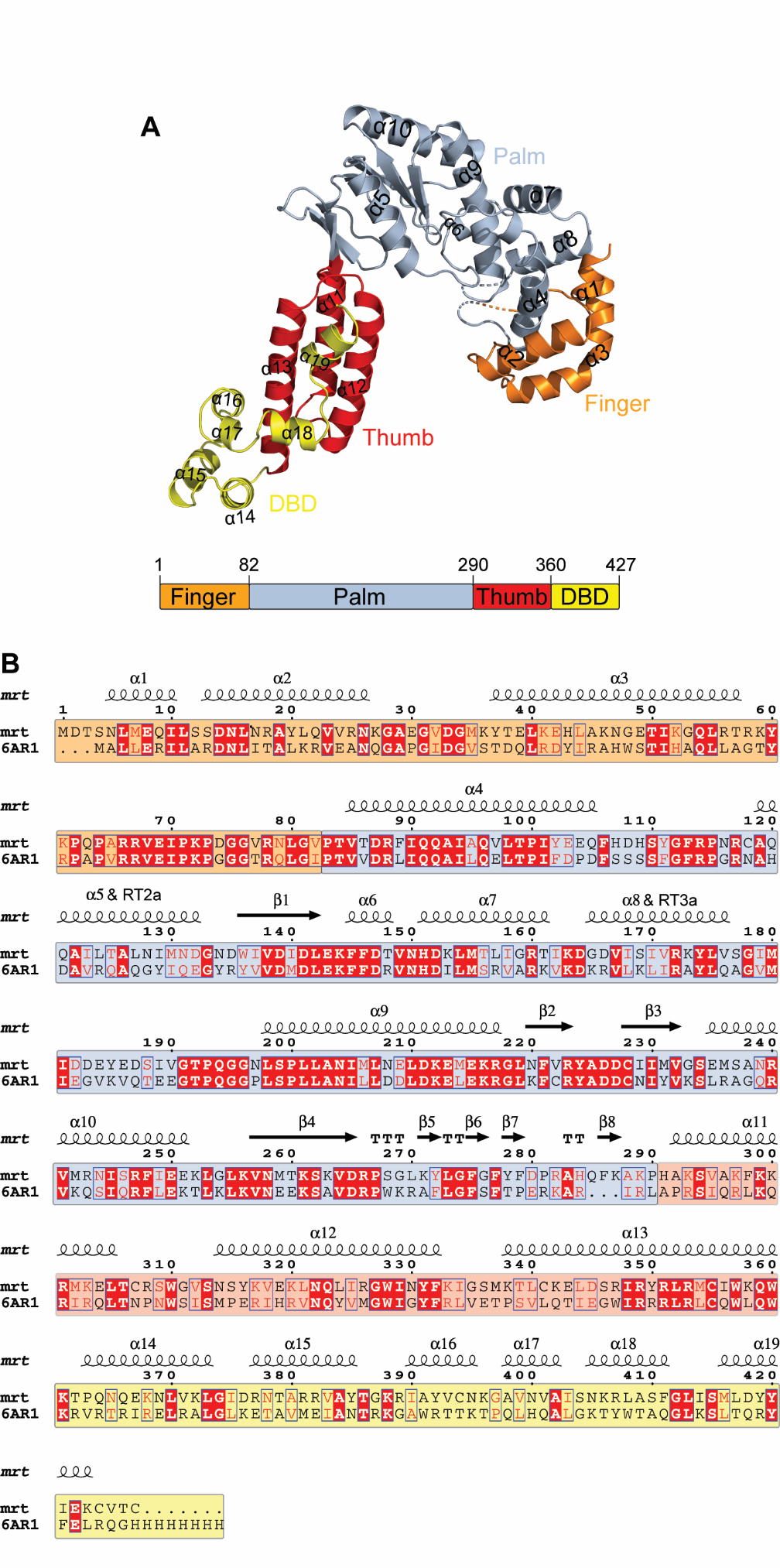
**

**Fig. S5. Structure of MarathonRT**

**(A).** Model of MarathonRT, the maturase protein, built into the cryoEM map density. MarathonRT consists of a finger, palm, thumb and DNA binding domain (DBD). The α-helices are numbered consecutively from 1-19. **(B)**. Sequence alignment (done with ClustalOmega (*62*)) of MarathonRT against the group IIC GscII maturase (PDB: 6AR1). A red background denotes positions that are identical between sequences and a white background, with red lettering denotes similar amino acid content. The secondary structure of MarathonRT is depicted above the alignment. Graphics were prepared with ESPript 3.0 (*65*).

**
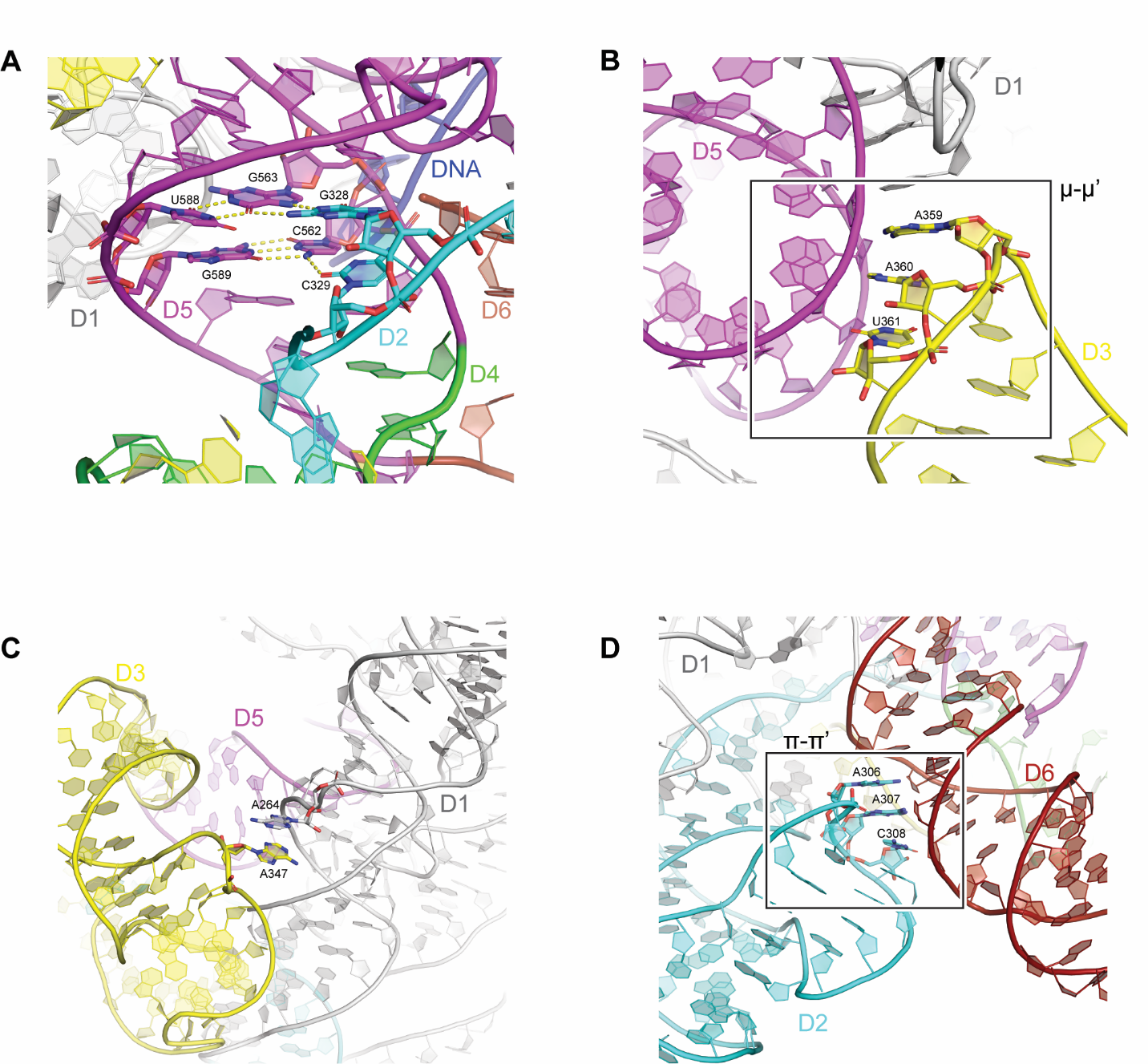
**

**Fig. S6. Tertiary Interactions within the holo-RNP**

**(A)**. Base triples formed by G328 and C329 of the J2/3 linker with G563-U588 and C562-G589 respectively. **(B)**. The μ-μ’ tertiary interaction between the pentaloop of D3 (A359, A360, U361) and the minor groove of the D5 helix. **(C).** An A-stacking interaction between A264 of D1 and A347 of D3 **(D).** The π-π’ tertiary interaction between D2b (A306, A307, C308) and the basal portion of the D6 helix.

**
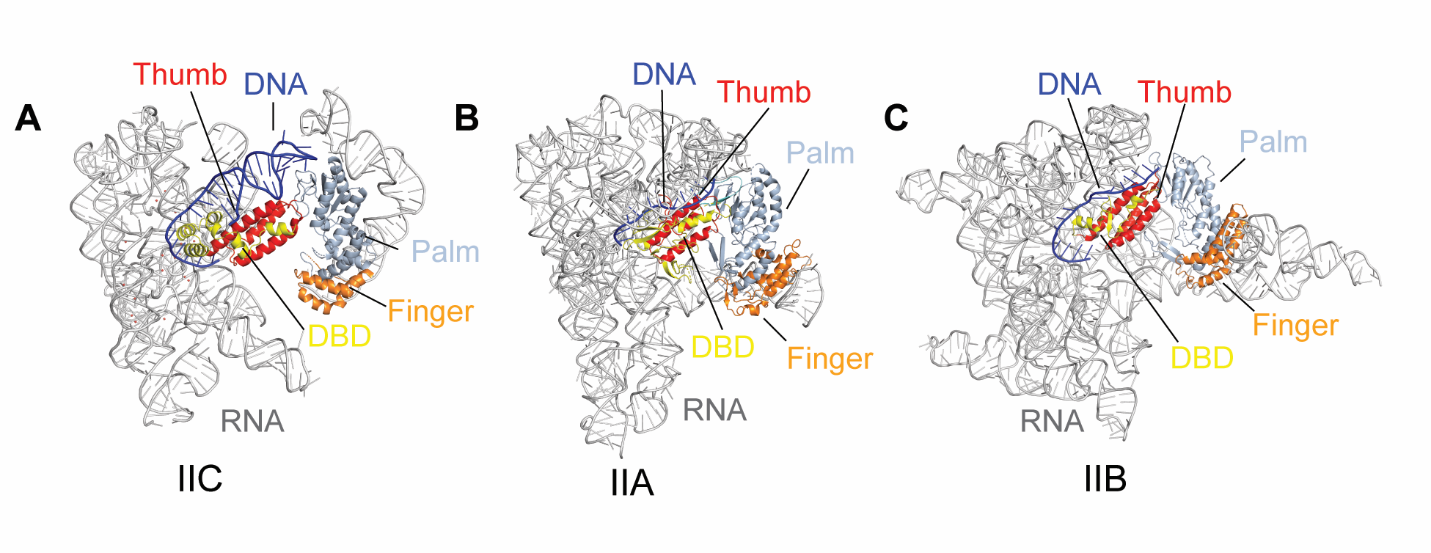
Fig. S7. Maturase Positioning in Group II RNPs**

**(A-C).** The maturase is coordinated by D4a in group II RNPs. The fingers, palm, thumb, DBD and DNA/mRNA substrate are indicated in orange, light blue, red, yellow, and dark blue respectively. The maturase thumb exclusively coordinates the DNA substrate and is not observed to interact directly with the EBS sequences in IIA and IIB RNPs. In IIA and IIB intron RNPs a linear DNA substrate is sandwiched between the protein and intron EBS loops, whereas in IIC intron RNPs, the DNA sits along the maturase thumb and DBD, allowing the protein to approach the substrate recognition loops within the ribozyme core. **(A).** Class IIC intron. **(B).** Class IIA intron (PDB: 5G2X). **(C).** Class IIB intron (PDB: 6ME0).

**
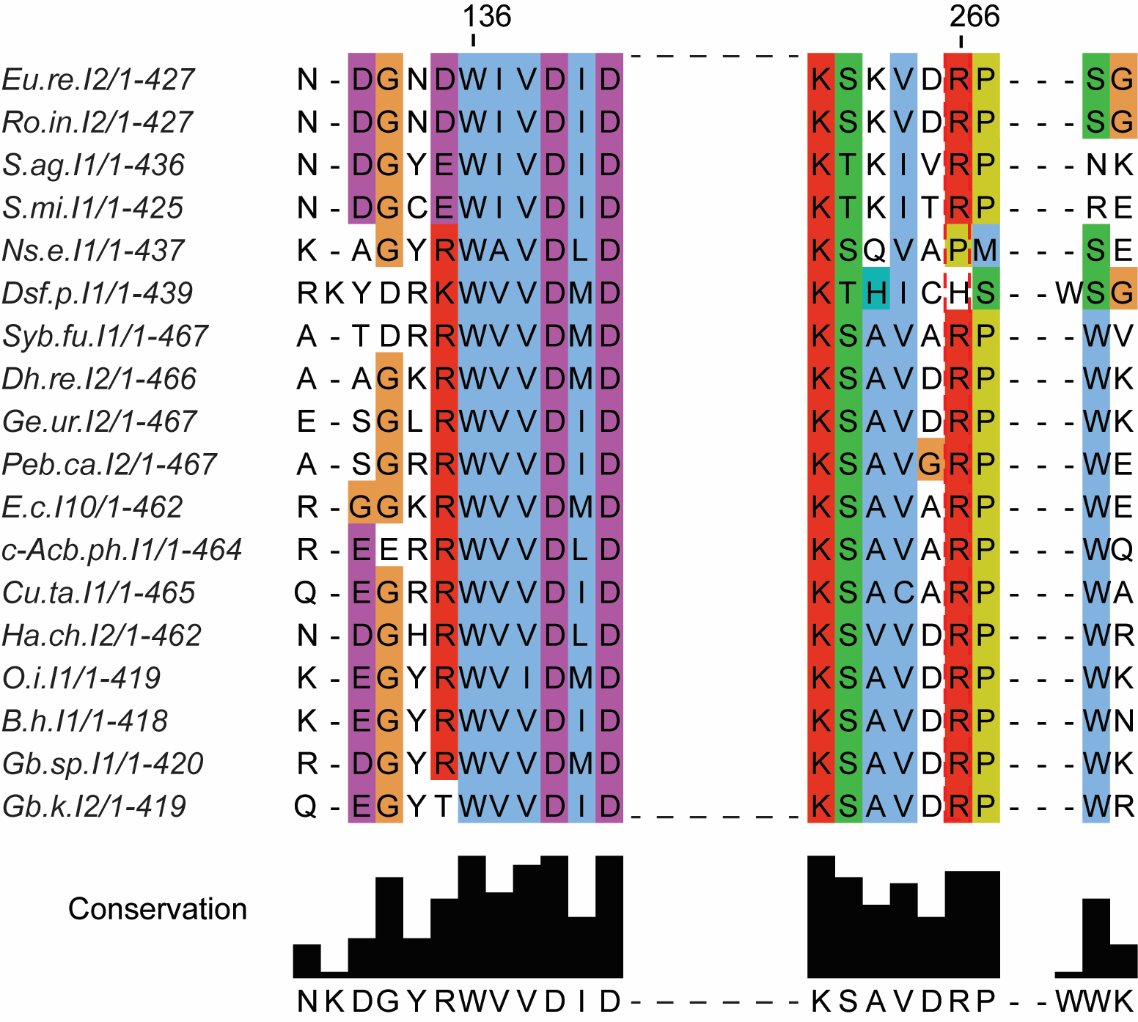
**

**Fig. S8. Conservation of the Protein Residues in IIC Maturases**

Sequence alignment of bacterial group IIC IEPs. The regions surrounding the two amino acids, W136 and R266, both within the palm domain, involved in the arginine-π stacking sandwich with DNA are shown.


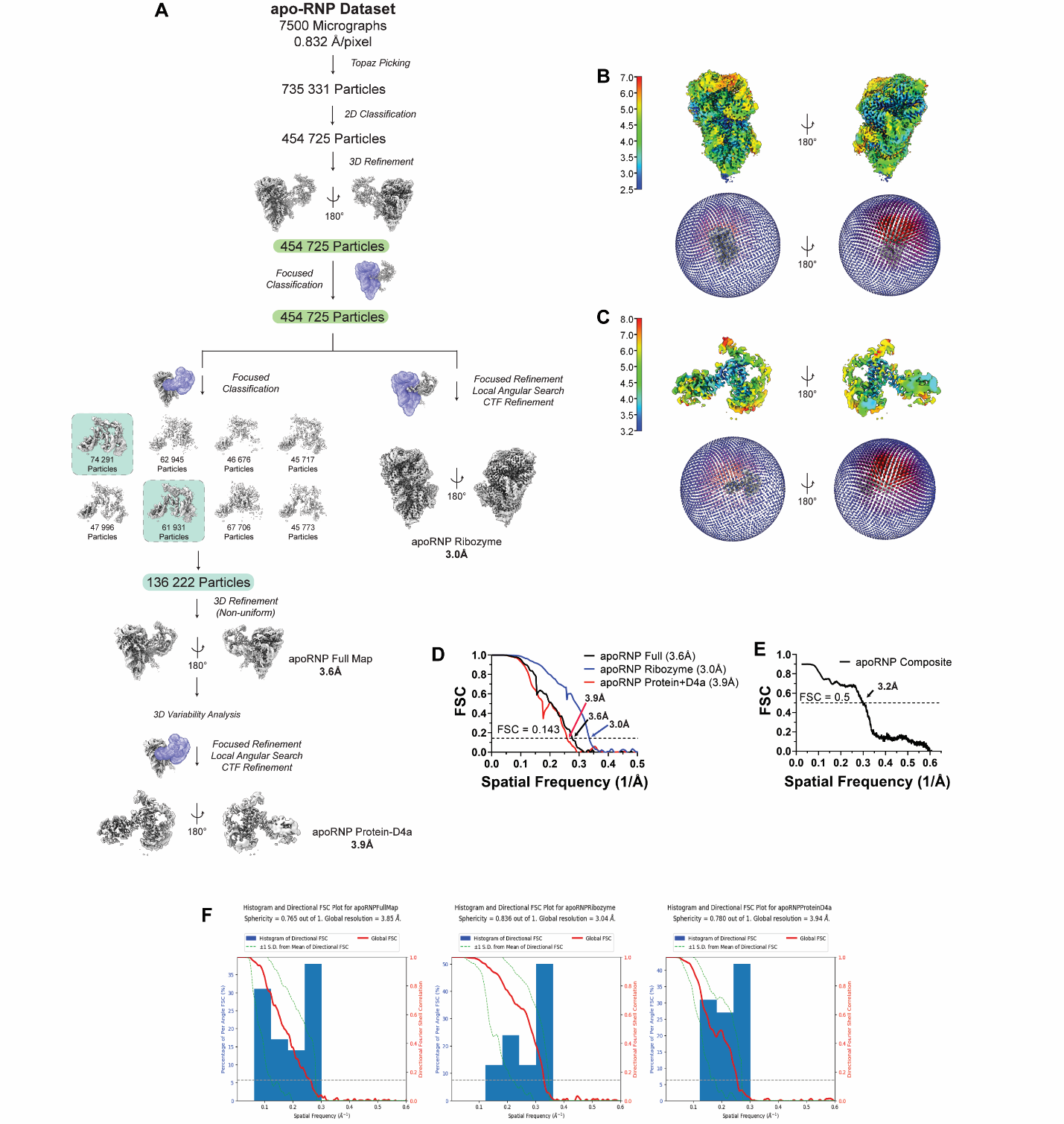
**Fig. S9. CryoEM workflow of the lariat-maturase apo-RNP complex.**

**(A).** CryoEM data processing workflow of the lariat-maturase complex (details in methods). **(B-C).** Local resolution map and particle distribution of **(B)** the apo-RNP ribozyme map and **(C)**. the apo-RNP protein-D4a map. **(D).** FSC curves with the gold standard threshold of 0.143 for all three apo-RNP complex maps. **(E).** FSC curve for the model refined against the apo-RNP composite map. **(F).** 3D FSC analysis of the apo-RNP full reconstruction, and the focused refined apo-RNP ribozyme reconstruction and the apo-RNP protein-D4a reconstruction.

**
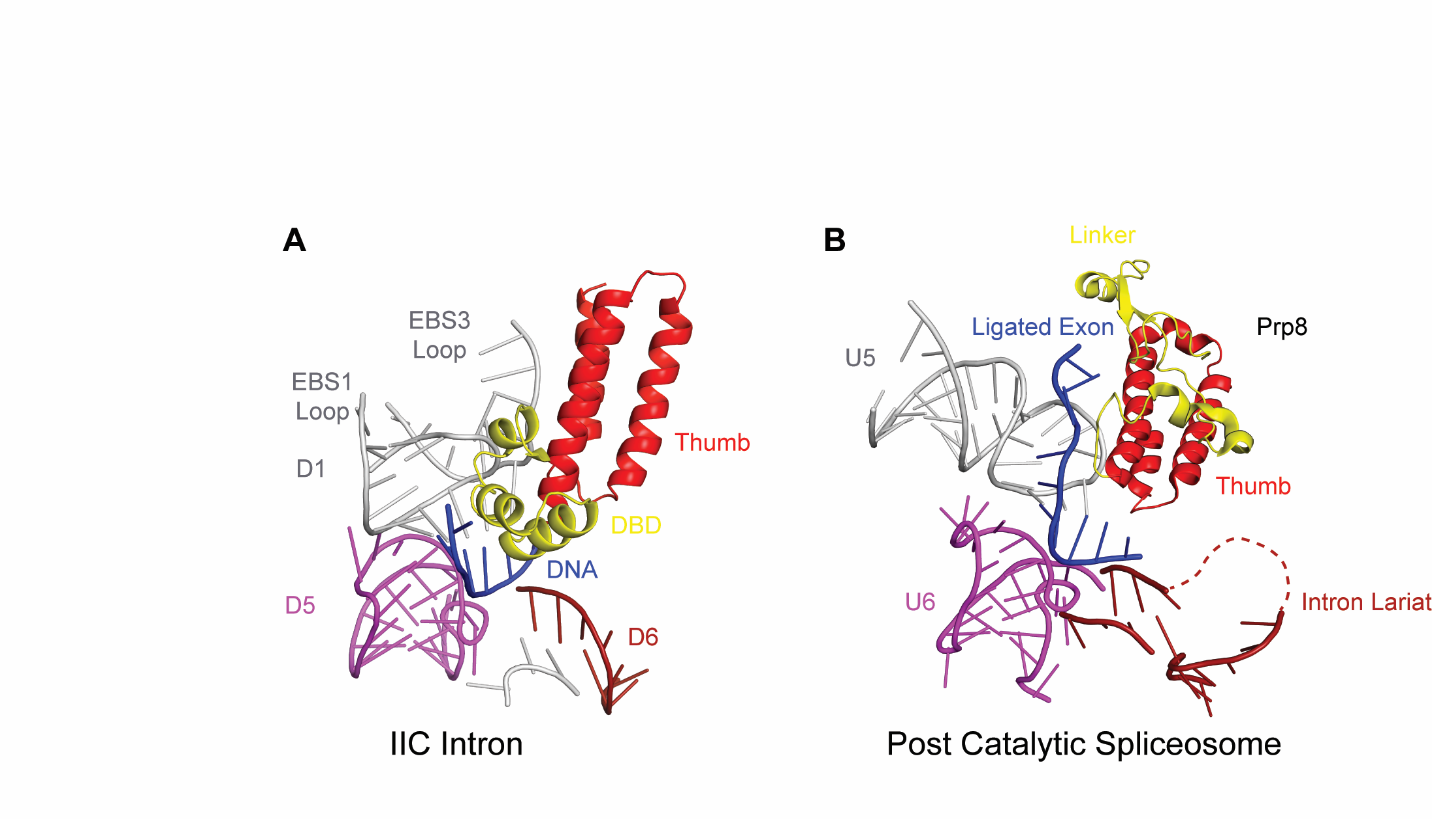
**

**Fig. S10. Parallels with Post Catalytic Human Spliceosome**

**(A-B).** Comparison of the positioning of the substrate recognition loops relative to the protein thumb and DBD/linker regions within the active site. **(A).** Group IIC intron. **(B).** Human post-catalytic spliceosome P-complex (PDB: 6QDV). The Prp8 thumb is angled inwards toward the U5 recognition loop.

**
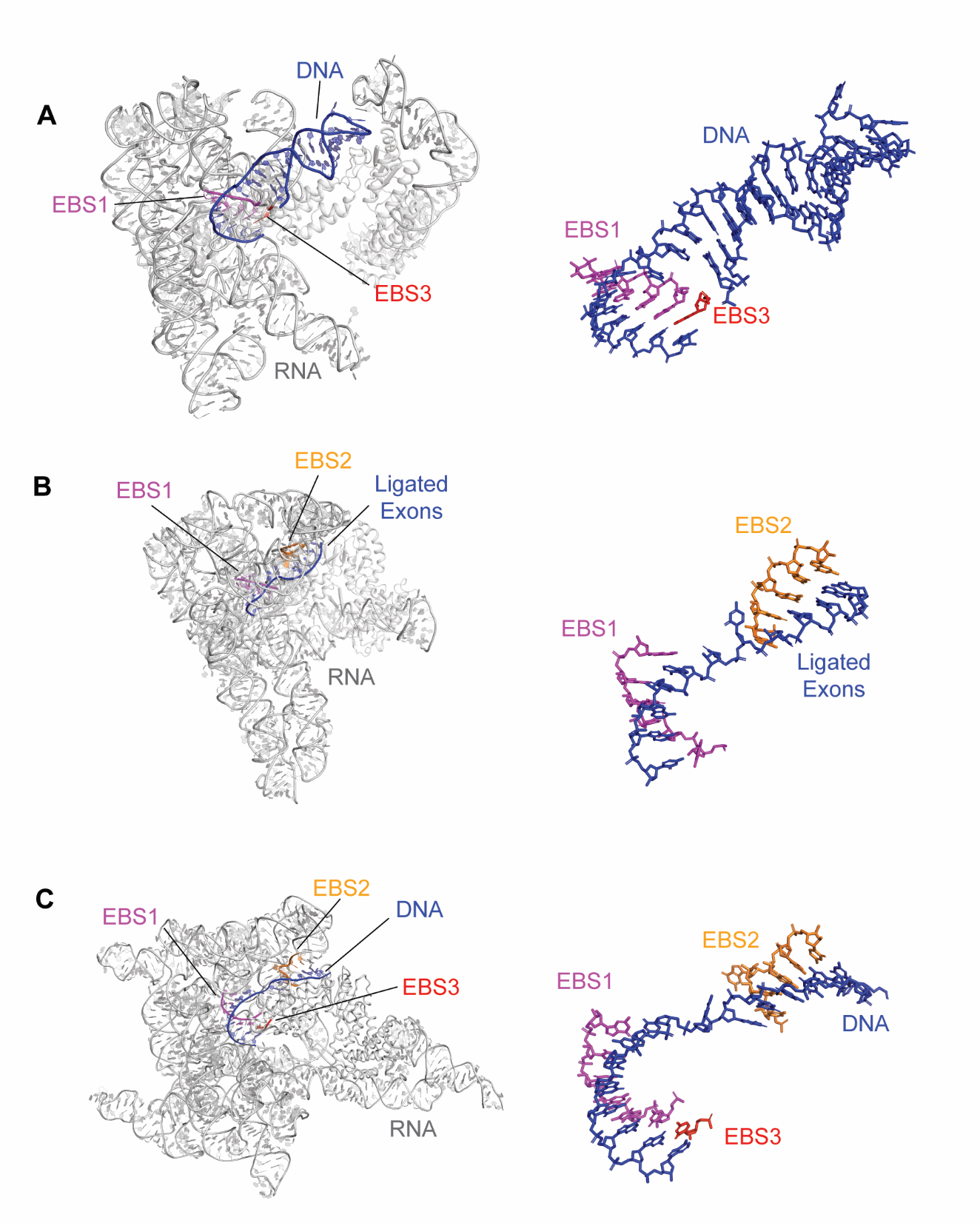
**

**Fig. S11. Mimicry of the DNA Structural Motif**

**(A-C).** EBS-IBS interactions of three different classes of group II introns. DNA/mRNA substrate is shown in blue. EBS1 nucleotides are colored magenta. EBS3 nucleotides are colored red. EBS2 interactions are in orange. **(A).** Class IIC intron. **(B).** Class IIA intron (PDB: 5G2X). **(C).** Class IIB Intron (PDB: 6ME0).

**
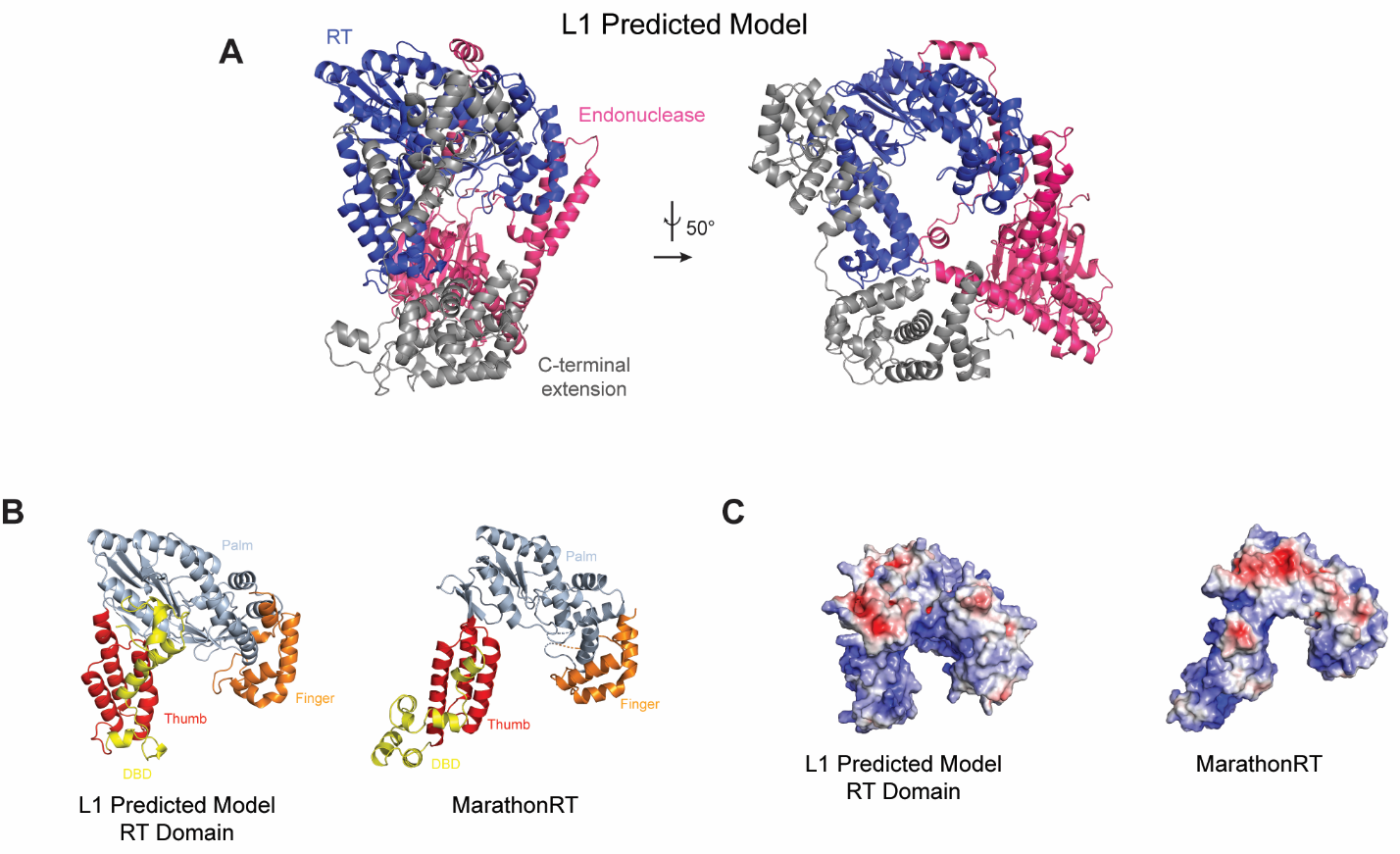
**

**Fig. S12. Predicted Structure of L1 ORF2p**

**(A).** Domain organization of L1 ORF2p. **(B).** Comparison of the RT domain of L1 ORF2p with MarathonRT. **(C).** Surface charges of the RT domain of L1 ORF2p (left) and MarathonRT (right). Blue indicates a positive charge while red indicates a negative charge.

**Table S1. Cryo-EM Data Collection, Refinement, and Validation Statistics.**

|  | **HoloRNP Full Map** | **HoloRNP Left Map** | **Holo-RNP Right Map** | **Holo-RNP Composite Map** | **Apo-RNP Full Map** | **Apo-RNP Ribozyme Map** | **Apo-RNP Protein-D4a Map** | **Apo-RNP Composite Map** | |
| --- | --- | --- | --- | --- | --- | --- | --- | --- | --- |
| **EMDB** | **26550** | **26550** | **26550** | **26550** | **26549** | **26549** | **26549** | **26549** | |
| **PDB** | **7UIN** | **7UIN** | **7UIN** | **7UIN** | **7UIM** | **7UIM** | **7UIM** | **7UIM** | |
|  |  |  |  |  |  |  |  |  | |
| **Data Collection and Processing** | | | | | | | | |  |
| **Voltage (kV)** | 300 | 300 | 300 | 300 | 300 | 300 | 300 | 300 | |
| **Symmetry Imposed** | C1 | C1 | C1 | C1 | C1 | C1 | C1 | C1 | |
| **Magnification** | 105,000 | 105,000 | 105,000 | 105,000 | 105,000 | 105,000 | 105,000 | 105,000 | |
| **Defocus Range (µm)** | 0.5-2.0 | 0.5-2.0 | 0.5-2.0 | 0.5-2.0 | 1.0-2.5 | 1.0-2.5 | 1.0-2.5 | 1.0-2.5 | |
| **Total Dose (e-/A^2^)** | 50.85 | 50.85 | 50.85 | 50.85 | 50.27 | 50.27 | 50.27 | 50.27 | |
| **Pixel Size (Å)** | 0.832 | 0.832 | 0.832 | 0.832 | 0.832 | 0.832 | 0.832 | 0.832 | |
| **Micrographs Collected** | 14 464 | 14 464 | 14 464 | 14 464 | 8 005 | 8 005 | 8 005 | 8 005 | |
| **Micrographs Processed** | 13 592 | 13 592 | 13 592 | 13 592 | 7 416 | 7 416 | 7 416 | 7 416 | |
| **Initial Particles** | 5 729 940 | 5 729 940 | 5 729 940 | 5 729 940 | 735 331 | 735 331 | 735 331 | 735 331 | |
| **Particles After 2D Classification** | 4 503 395 | 4 503 395 | 4 503 395 | 4 503 395 | 454 725 | 454 725 | 454 725 | 454 725 | |
| **Particles for 3D Refinement** | 300 344 | 300 344 | 300 344 | 300 344 | 136 222 | 454 725 | 136 222 | 454 725 | |
|  |  |  |  |  |  |  |  |  | |
| **Refinement** | | | | | | | | |  |
| **Map Resolution** | 2.8 | 2.7 | 3.0 |  | 3.6 | 3.0 | 3.9 |  | |
| **FSC Threshold** | 0.143 | 0.143 | 0.143 |  | 0.143 | 0.143 | 0.143 |  | |
| **Map sharpening B-factor (A^2^)** | -72.1 | -67.8 | -96 |  | -76 | -79.8 | -140.9 |  | |
| **FSC Map to Model** | 2.9 |  |  |  |  |  |  | 3.2 | |
| **FSC Threshold** | 0.5 |  |  |  |  |  |  | 0.5 | |
|  |  |  |  |  |  |  |  |  | |
| **Model Composition** | | | | | | | | |  |
| **Non-hydrogen atoms** | 15 585 |  |  |  |  |  |  | 14768 | |
| **Protein residues** | 387 |  |  |  |  |  |  | 387 | |
| **RNA bases** | 541 |  |  |  |  |  |  | 541 | |
| **DNA bases** | 37 |  |  |  |  |  |  | N/A | |
|  |  |  |  |  |  |  |  |  | |
| **B factors (A^2^)** |  |  |  |  |  |  |  |  | |
| **Protein** | 23.35 |  |  |  |  |  |  | 82.64 | |
| **Nucleotide** | 52.01 |  |  |  |  |  |  | 70.95 | |
|  |  |  |  |  |  |  |  |  | |
| **R.m.s deviation** | | | | | | | | |  |
| **Bond length (Å)** | 0.01 |  |  |  |  |  |  | 0.005 | |
| **Bond angles (⁰)** | 1.206 |  |  |  |  |  |  | 0.866 | |
|  |  |  |  |  |  |  |  |  | |
| **Validation** | | | | | | | | |  |
| **Molprobity score** | 1.86 |  |  |  |  |  |  | 1.64 | |
| **Clashscore** | 4.39 |  |  |  |  |  |  | 7.72 | |
|  |  |  |  |  |  |  |  |  | |
| **Ramachandran plot** | | | | | | | | |  |
| **Outliers (%)** | 0.52 |  |  |  |  |  |  | 0.00 | |
| **Allowed (%)** | 3.67 |  |  |  |  |  |  | 3.41 | |
| **Favored (%)** | 95.80 |  |  |  |  |  |  | 96.59 | |

**References:**

46. A. Punjani, J. L. Rubinstein, D. J. Fleet, M. A. Brubaker, *Nat Methods* **14**, 290-296 (2017).

47. A. Punjani, H. Zhang, D. J. Fleet, *Nat Methods* **17**, 1214-1221 (2020).

48. T. Bepler *et al.*, *Res Comput Mol Biol* **10812**, 245-247 (2018).

49. A. Punjani, D. J. Fleet, *J Struct Biol* **213**, 107702 (2021).

50. Y. Z. Tan *et al.*, *Nat Methods* **14**, 793-796 (2017).

51. T. D. Goddard, C. C. Huang, T. E. Ferrin, *J Struct Biol* **157**, 281-287 (2007).

52. E. F. Pettersen *et al.*, *J Comput Chem* **25**, 1605-1612 (2004).

53. P. Emsley, B. Lohkamp, W. G. Scott, K. Cowtan, *Acta Crystallogr D Biol Crystallogr* **66**, 486-501 (2010).

54. R. T. Kidmose *et al.*, *IUCrJ* **6**, 526-531 (2019).

55. P. D. Adams *et al.*, *Acta Crystallogr D Biol Crystallogr* **66**, 213-221 (2010).

56. P. V. Afonine *et al.*, *Acta Crystallogr D Struct Biol* **74**, 531-544 (2018).

57. D. Liebschner *et al.*, *Acta Crystallogr D Struct Biol* **75**, 861-877 (2019).

58. E. Krissinel, K. Henrick, *J Mol Biol* **372**, 774-797 (2007).

59. E. F. Pettersen *et al.*, *Protein Sci* **30**, 70-82 (2021).

60. A. R. Gruber, R. Lorenz, S. H. Bernhart, R. Neubock, I. L. Hofacker, *Nucleic Acids Res* **36**, W70-74 (2008).

61. X. J. Lu, W. K. Olson, *Nat Protoc* **3**, 1213-1227 (2008).

62. F. Sievers, D. G. Higgins, *Methods Mol Biol* **1079**, 105-116 (2014).

63. A. M. Waterhouse, J. B. Procter, D. M. Martin, M. Clamp, G. J. Barton, *Bioinformatics* **25**, 1189-1191 (2009).

64. P. Z. Johnson, W. K. Kasprzak, B. A. Shapiro, A. E. Simon, *RNA Biol* **16**, 1667-1671 (2019).

65. P. Gouet, E. Courcelle, *Bioinformatics* **18**, 767-768 (2002).

**Movie S1.**

D5 active site helix with density shown as mesh.

**Movie S2.**

CryoEM reconstruction of the holoenzyme complex.

**Movie S3.**

Catalytic elements of the ribozyme active site.

**Movie S4.**

Interactions with the structured DNA target.

**Movie S5.**

3D variability analysis of the holoRNP complex.

**Movie S6.**

3D variability analysis of the apoRNP complex.

**Materials Design Analysis Reporting (MDAR)**

**Checklist for Authors**

The MDAR framework establishes a minimum set of requirements in transparent reporting applicable to studies in the life sciences (see Statement of Task: doi:10.31222/osf.io/9sm4x.). The MDAR checklist is a tool for authors, editors, and others seeking to adopt the MDAR framework for transparent reporting in manuscripts and other outputs. Please refer to the MDAR Elaboration Document for additional context for the MDAR framework.

**For all that apply, please note where in the manuscript the required information is provided.**

**Materials:**

| **Newly created materials** | **indicate where provided: page no/section/legend)** | **n/a** |
| --- | --- | --- |
| The manuscript includes a dedicated "materials availability statement" providing transparent disclosure about availability of newly created materials including details on how materials can be accessed and describing any restrictions on access. |  | N/A |
|  |  |  |
| **Antibodies** | **indicate where provided: page no/section/legend)** | **n/a** |
| For commercial reagents, provide supplier name, catalogue number and [RRID](https://scicrunch.org/resources), if available. |  | N/A |
|  |  |  |
| **DNA and RNA sequences** | **indicate where provided: page no/section/legend)** | **n/a** |
| **Short novel DNA or RNA including primers, probes:** Sequences should be included or deposited in a public repository. | Yes. Supplementary Materials/ Materials and Methods |  |
|  |  |  |
| **Cell materials** | **indicate where provided: page no/section/legend** | **n/a** |
| **Cell lines:** Provide species information, strain. Provide accession number in repository **OR** supplier name, catalog number, clone number, **OR** RRID. |  | N/A |
| **Primary cultures:** Provide species, strain, sex of origin, genetic modification status. |  | N/A |
|  |  |  |
| **Experimental animals** | **indicate where provided: page no/section/legend)** | **n/a** |
| **Laboratory animals or Model organisms:** Provide species, strain, sex, age, genetic modification status. Provide accession number in repository **OR** supplier name, catalog number, clone number, **OR** RRID. |  | N/A |
| **Animal observed in or captured from the field:** Provide species, sex, and age where possible. |  | N/A |
|  |  |  |
| **Plants and microbes** | **indicate where provided: page no/section/legend)** | **n/a** |
| **Plants:** provide species and strain, ecotype and cultivar where relevant, unique accession number if available, and source (including location for collected wild specimens). |  | N/A |
| **Microbes:** provide species and strain, unique accession number if available, and source. |  | N/A |
|  |  |  |
| **Human research participants** | **indicate where provided: page no/section/legend) or state if these demographics were not collected** | **n/a** |
| If collected and within the bounds of privacy constraints report on age, sex and gender or ethnicity for all study participants. |  | N/A |

**Design:**

| **Study protocol** | **indicate where provided: page no/section/legend)** | **n/a** |
| --- | --- | --- |
| If study protocol has been pre-registered, provide DOI. For clinical trials, provide the trial registration number **OR** cite DOI. |  | N/A |
|  |  |  |
| **Laboratory protocol** | **indicate where provided: page no/section/legend)** | **n/a** |
| Provide DOI **OR** other citation details if detailed step-by-step protocols are available. |  | N/A |
|  |  |  |
| **Experimental study design (statistics details)** | | |
| **For in vivo studies:** State whether and how the following have been done | **indicate where provided: page no/section/legend. If it could have been done, but was not, write not done** | **n/a** |
| Sample size determination |  | N/A |
| Randomisation |  | N/A |
| Blinding |  | N/A |
| Inclusion/exclusion criteria |  | N/A |
|  |  |  |
| **Sample definition and in-laboratory replication** | **indicate where provided: page no/section/legend** | **n/a** |
| State number of times the experiment was replicated in laboratory. |  | N/A |
| Define whether data describe technical or biological replicates. |  | N/A |
|  |  |  |
| **Ethics** | **indicate where provided: page no/section/legend** | **n/a** |
| **Studies involving human participants:** State details of authority granting ethics approval (IRB or equivalent committee(s), provide reference number for approval. |  | N/A |
| **Studies involving experimental animals:** State details of authority granting ethics approval (IRB or equivalent committee(s), provide reference number for approval. |  | N/A |
| **Studies involving specimen and field samples:** State if relevant permits obtained, provide details of authority approving study; if none were required, explain why. |  | N/A |
|  |  |  |
| **Dual Use Research of Concern (DURC)** | **indicate where provided: page no/section/legend** | **n/a** |
| If study is subject to dual use research of concern regulations, state the authority granting approval and reference number for the regulatory approval. |  | N/A |

**Analysis:**

| **Attrition** | **indicate where provided: page no/section/legend** | **n/a** |
| --- | --- | --- |
| Describe whether exclusion criteria were preestablished. Report if sample or data points were omitted from analysis. If yes report if this was due to attrition or intentional exclusion and provide justification. |  | N/A |
|  |  |  |
| **Statistics** | **indicate where provided: page no/section/legend** | **n/a** |
| Describe statistical tests used and justify choice of tests. |  | N/A |
|  |  |  |
| **Data availability** | **indicate where provided: page no/section/legend** | **n/a** |
| For newly created and reused datasets, the manuscript includes a data availability statement that provides details for access or notes restrictions on access. | The cryo-EM maps and model coordinates have been deposited to the Electron Microscopy Data Band (EMDB) and Protein Data Bank (PDB) respectively. |  |
| If newly created datasets are publicly available, provide accession number in repository **OR** DOI **OR** URL and licensing details where available. | EMDB: 26549 (apo), 26550 (holo)  PDB ID: 7UIM (apo), 7UIN (holo) |  |
| If reused data is publicly available provide accession number in repository **OR** DOI **OR** URL, **OR** citation. |  | N/A |
|  |  |  |
| **Code availability** | **indicate where provided: page no/section/legend** | **n/a** |
| For all newly generated custom computer code/software/mathematical algorithm or re-used code essential for replicating the main findings of the study, the manuscript includes a data availability statement that provides details for access or notes restrictions. |  |  |
| If newly generated code is publicly available, provide accession number in repository, **OR** DOI **OR** URL and licensing details where available. State any restrictions on code availability or accessibility. |  | N/A |
| If reused code is publicly available provide accession number in repository **OR** DOI **OR** URL, **OR** citation. |  | N/A |

**Reporting**

MDAR framework recommends adoption of discipline-specific guidelines, established and endorsed through community initiatives. Journals have their own policy about requiring specific guidelines and recommendations to complement MDAR.

| **Adherence to community standards** | **indicate where provided: page no/section/legend** | **n/a** |
| --- | --- | --- |
| State if relevant guidelines (e.g., ICMJE, MIBBI, ARRIVE) have been followed, and whether a checklist (e.g., CONSORT, PRISMA, ARRIVE) is provided with the manuscript. |  | N/A |
